# Polyglutamine (PolyQ) Diseases: Navigating the Landscape of Neurodegeneration

**DOI:** 10.1021/acschemneuro.4c00184

**Published:** 2024-07-12

**Authors:** Rumiana Tenchov, Janet M. Sasso, Qiongqiong Angela Zhou

**Affiliations:** CAS, a division of the American Chemical Society, Columbus, Ohio 43210, United States

**Keywords:** polyglutamine, CAG repeat, Huntington’s disease, spinocerebellar ataxia, dentatorubral pallidoluysian atrophy, spinal and bulbar muscular atrophy, pathogenesis, protein misfolding, protein aggregation

## Abstract

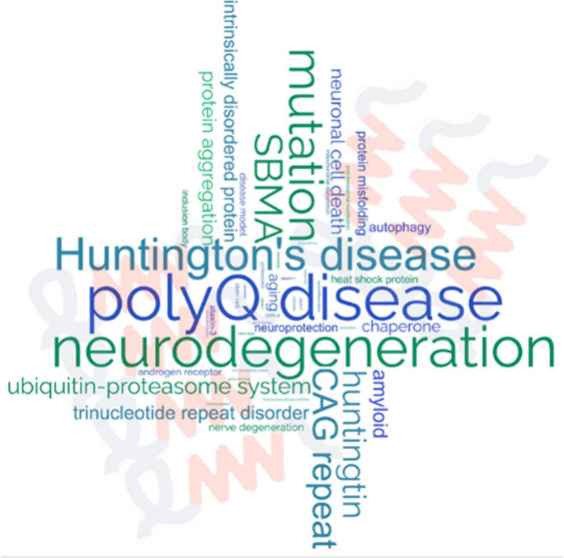

Polyglutamine (polyQ) diseases are a group of inherited neurodegenerative disorders caused by expanded cytosine-adenine-guanine (CAG) repeats encoding proteins with abnormally expanded polyglutamine tract. A total of nine polyQ disorders have been identified, including Huntington’s disease, six spinocerebellar ataxias, dentatorubral pallidoluysian atrophy (DRPLA), and spinal and bulbar muscular atrophy (SBMA). The diseases of this class are each considered rare, yet polyQ diseases constitute the largest group of monogenic neurodegenerative disorders. While each subtype of polyQ diseases has its own causative gene, certain pathologic molecular attributes have been implicated in virtually all of the polyQ diseases, including protein aggregation, proteolytic cleavage, neuronal dysfunction, transcription dysregulation, autophagy impairment, and mitochondrial dysfunction. Although animal models of polyQ disease are available helping to understand their pathogenesis and access disease-modifying therapies, there is neither a cure nor prevention for these diseases, with only symptomatic treatments available. In this paper, we analyze data from the CAS Content Collection to summarize the research progress in the class of polyQ diseases. We examine the publication landscape in the area in effort to provide insights into current knowledge advances and developments. We review the most discussed concepts and assess the strategies to combat these diseases. Finally, we inspect clinical applications of products against polyQ diseases with their development pipelines. The objective of this review is to provide a broad overview of the evolving landscape of current knowledge regarding the class of polyQ diseases, to outline challenges, and evaluate growth opportunities to further efforts in combating the diseases.

## Overview of the Neurodegenerative Diseases

Neurodegenerative diseases are a class of neurological disorders, which critically harm the lives of millions of people worldwide. They are characterized by progressive loss of neurons in the nervous system. The collapse of the neural networks associated with loss of neurons, which are unable to effectively renew because of their terminally differentiated postmitotic nature, result in the failure of the core communicative connections, leading to impaired memory, cognition, behavior, sensory, and/or motoric performance.^1^ Neurodegenerative diseases are complex disorders in which multiple factors such as genomic, epigenomic, cerebrovascular, metabolic, environmental, and others, converge to outline a progressive neurodegenerative phenotype. Concomitant with the rise in longevity over the past decades, there has been an escalation in the incidence of neurodegenerative disorders.^2^

**Figure 1:**
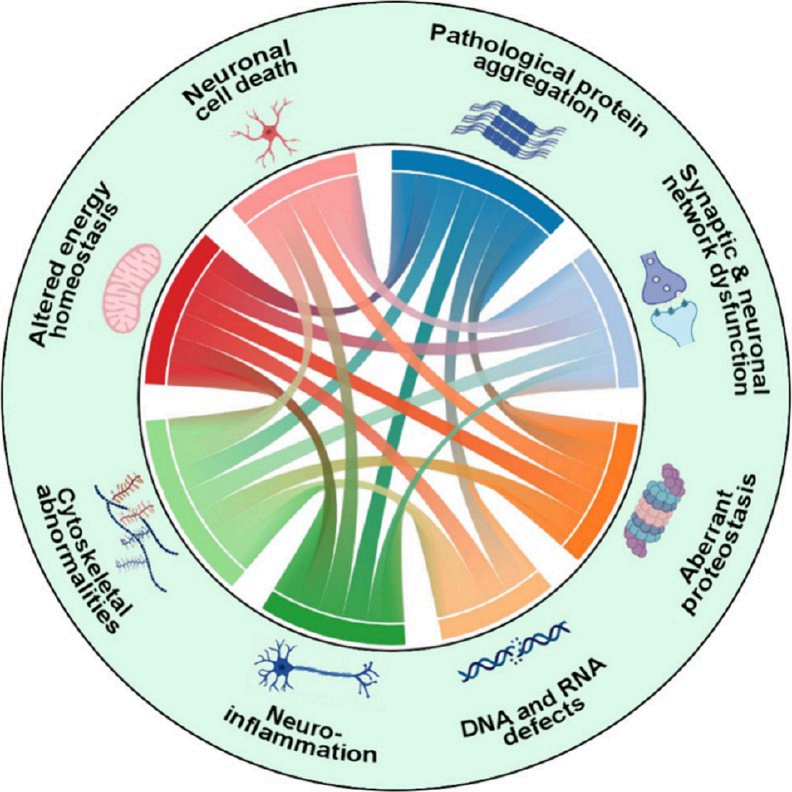
Hallmarks of neurodegenerative diseases and relationships between them.

Although neurodegenerative diseases are characteristically defined by particular protein accumulations and anatomic vulnerability, they share multiple fundamental processes associated with the progressive neuronal dysfunction and death, such as proteotoxic stress and its associated dysfunctions in ubiquitin–proteasome and autophagosome/lysosome systems, oxidative stress, programmed cell death, and neuroinflammation.^3^ It has been agreed that neurodegenerative diseases are defined by a set of common attributes including: pathological protein aggregation, synaptic and neuronal network dysfunction, aberrant proteostasis, cytoskeletal abnormalities, altered energy metabolism, DNA and RNA defects, inflammation, and neuronal cell death (Figure 1).^1−4^

Distinctive protein aggregation is a key pathological hallmark of a variety of neurodegenerative diseases and is related to virtually all other neurodegeneration attributes.^5,6^ Thus, protein aggregates are believed to trigger inflammatory responses in the brain. Microglia, the brain’s immune cells, become activated and can release inflammatory molecules that damage neurons. This neuroinflammation further contributes to neuronal dysfunction and death. Protein aggregates can interfere with the communication between neurons at synapses. This disrupts neural circuits and contributes to cognitive decline, memory loss, and movement problems seen in neurodegenerative diseases. The balance of protein synthesis, folding, and degradation, known as proteostasis, is disturbed by protein aggregation. This can lead to further misfolding and accumulation of proteins. Protein aggregates can interfere with the cytoskeleton, the structure that maintains cell shape and facilitates intracellular transport, contributing to neuronal dysfunction. The presence of protein aggregates can affect mitochondrial function and energy production, which is vital for neuron survival. Aggregated proteins may also be involved in the disruption of genetic material processing, leading to further cellular stress and damage. Ultimately, the presence of protein aggregates can lead to cell death, which is a definitive feature of neurodegenerative diseases. It is important to note that the exact cause-and-effect relationship between neurodegenerative hallmarks is still being investigated. For example, while protein aggregation is a defining feature, it is not entirely clear if it is the initial trigger, or a consequence of other cellular processes gone awry. Understanding these relationships is crucial for developing targeted therapies that can address multiple aspects of these complex diseases.

Pathological protein aggregation often serves for diagnosis and disease classification. In neurodegenerative diseases, symptoms generally reflect the disruption of specific neuronal networks, and synaptic failure is an early event preceding neuronal loss, since neuronal network function requires precise synaptic function.^7,8^

The accumulation of ubiquitinated, aggregated proteins in many neurodegenerative diseases indicates altered, abnormal proteostasis. Abundant abnormal aggregates of cytoskeletal proteins are neuropathological signatures of many neurodegenerative diseases and represent another hallmark of neurodegeneration related to all other degeneration attributes.^9,10^ Mitochondrial dysfunction is repetitively involved in the pathogenesis of diverse neurodegenerative diseases. It is associated with certain molecular and cellular defects, whose impact at different levels of multifactorial nature including the calcium and iron homeostasis, energetic balance and/or oxidative stress.^11,12^

The accumulation of DNA damage and defects in RNA metabolism have been allocated a key role in a variety of neurodegenerative diseases, as far as the cellular genome and transcriptome are vulnerable to spontaneous decay and damage by multiple intracellular or environmental agents.^13,14^ Neuroinflammation is a pathological hallmark of a wide range of neurodegenerative diseases including Alzheimer’s disease, Parkinson’s disease, and amyotrophic lateral sclerosis.^2,15^

Certain inherent properties of neurons may make them particularly vulnerable to cell death in neurodegenerative diseases, including, e.g., their postmitotic nature resulting in gradual accumulation of age-associated damage and their high energy requirements.^1^ All other hallmarks of neurodegeneration individually and collectively contribute to neuronal cell loss.

**Figure 2:**
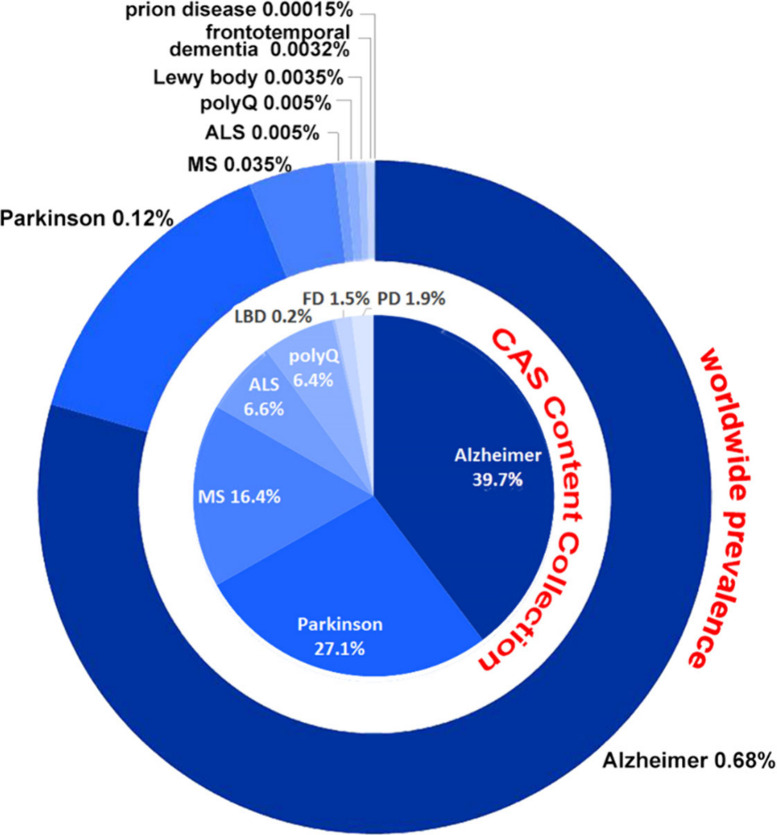
Worldwide prevalence of major neurodegenerative diseases (outer circle) and distribution of the number of documents related to those diseases in the CAS Content Collection^20^ (inner circle); LBD, Lewy body dementia; FD, frontotemporal dementia; PD, prion disease.

Neurodegenerative diseases include multiple disorders, such as Alzheimer’s disease, Parkinson’s disease, amyotrophic lateral sclerosis, multiple sclerosis, polyQ diseases including Huntington’s disease, spinocerebellar ataxias, and others.^16−19^ The worldwide prevalence of several notable neurodegenerative diseases is depicted in Figure 2, along with the number of documents related to these diseases in the CAS Content Collection,^20^ the largest human-compiled multidisciplinary database of published documents and substances.

In this paper, we analyzed data from the CAS Content Collection to summarize the research advances in one distinctive class of neurodegenerative disorders–the polyQ diseases. The diseases of this class are each considered rare, yet polyQ diseases constitute the largest group of monogenic neurodegenerative disorders.^21^ We examine the publication landscape of recent research in the area of polyQ diseases in effort to provide insights into the knowledge advances and developments. We review the most discussed concepts and assess the state-of-the-art strategies to combat these diseases, based on the data from the CAS Content Collection. Finally, we inspect clinical applications of products against various polyQ diseases with their development pipelines.

The objective of this review is to provide a broad overview of the evolving landscape of current knowledge regarding the class of polyQ diseases, to outline challenges, and evaluate growth opportunities, to further efforts in solving the problems that remain. The novelty and merit of the article stem from the extensive, wide-ranging coverage of the most up-to-date scientific information accumulated in the CAS Content Collection allowing unique, unmatched breadth of landscape analysis and in-depth insights.

## PolyQ Diseases

Polyglutamine (polyQ) diseases are a group of rare neurodegenerative disorders characterized by the abnormal expansion of a **cytosine-adenine-guanine (CAG)** trinucleotide repeat, resulting in the production of proteins with an expanded polyglutamine tract. The repeat expansions in these diseases take place in gene coding regions and produce protein with an anomalous structure and function. The expanded CAG/polyQ domains are identified as the primary drivers of neurodegeneration in this disease family. They mainly affect the central nervous system and are associated with progressive degeneration, dysfunction, and death of specific populations of neurons.^22−26^

At present, a total of nine polyQ disorders have been identified: Huntington’s disease (HD); six spinocerebellar ataxias (SCA) types 1, 2, 3, 6, 7, 17; dentatorubral pallidoluysian atrophy (DRPLA); and spinal and bulbar muscular atrophy (SBMA) (Table 1).^26^ Since the initial identification of the genetic basis of the polyQ diseases,^27^ thorough research has been performed to characterize the molecular basis of these disorders. PolyQ diseases exhibit inverse correlation between the number of CAG repeats and the age of onset of disease. The proteins involved in different polyQ diseases differ in their function and location within the cell. Moreover, different brain regions and neuronal cell subtypes are affected in each polyQ disease. Yet, a common feature of polyQ diseases is the radical deterioration of neurons in the specific regions of the brain, which cause impairment in vital functioning. Clinically, polyQ diseases exhibit threshold occurrences. Progressive pathological features are noticed once repeat numbers exceed disease-specific limits. Moreover, trinucleotide tracts are unstable and increase their length upon transmission to the next generations. A higher number of repeats bring about an earlier and more severe disease phenotype.^28,29^

**Table 1:** Polyglutamine (polyQ) repeat expansion diseases^1,22,25,36−45^

**PolyQ disease**	**Causative gene**	**Gene locus**	**Mutated protein**	**Pathogenic Q repeat length**	**Normal Q repeat length**	**Normal function**	**Frequency worldwide**	**Neuropathology/affected regions**	**Clinical features/manifestations**	**Inclusions**
Huntingtin’s disease	HTT	4p16.3	Huntingtin	>39 (36–121)	6–34	scaffold protein; axonal transport, regulation of Ca signaling	3–7/100,000	striatum, cerebral cortex	chorea; progressive cognitive decline; psychiatric disorders	nucleus, cytoplasm
Spinocerebellar ataxias (SCAs)							1–4/100,000			
SCA1	ATXN1	6p22–23	Ataxin 1	>39 (39–88)	6–44	gene expression	1–2/100,000	cerebellum (Purkinje cells), brainstem, cerebral cortex, dentate nucleus	pyramidal signs, peripheral neuropathy, motor control decline	nucleus
SCA2	ATXN2	12q23–24	Ataxin 2	>31 (32–77)	15–24	RNA metabolism	unknown, common in Cuba -40/100,000	cerebellum (Purkinje cells), brainstem, cerebral cortex	slow eye movement, neuropathy, hyporeflexia, tremor, chorea	cytoplasm
SCA3(Machado-Joseph disease)	ATXN3	14q24–31	Ataxin 3	>55 (55–86)	13–36	deubiquitinase, poly ubiquitin editing enzyme	1–9/100,000	cerebellum (dentate nucleus), brainstem, basal ganglia, spinal cord	bulging eye, spasticity, fasciculations, sensory loss, amyotrophy, ataxia	nucleus
SCA6	CACNA1A	19p13	CACNA 1 P/Q-type α 1A	>19 (21–33)	4–19	Ca channel	0.02–0.31/100,000	cerebellum (dentate and inferior olivary nuclei, Purkinje cells)	pure cerebellar signs	nucleus(cytoplasm)
SCA7	ATXN7	3p12-p21	Ataxin 7	>37 (38–200)	4–35	STAGA coactivator complex	<1/100,000	cerebellum, retina, brainstem, visual cortex	retinal degeneration	nucleus
SCA17	TBP	6q27	TATA-binding protein	>43 (45–63)	25–42	transcription factor	0.16/100,000	cerebellum, striatum, cortex, substantia nigra	dystonia, dementia, involuntary movements, hyperreflexia	nucleus
Dentatorubral pallidoluysian atrophy (DRPLA) (Haw–River syndrome)	ATN1	12p13.31	Atrophin 1	>49 (49–88)	7–34	transcriptional corepressor	2.7/100,000	cerebellum (dentatorubral pathway), cerebral cortex, basal ganglia (globus pallidus, subthalamic nucleus)	ataxia, myoclonic epilepsy, choreoathetosis, cognitive deficits	nucleus
Spinal and bulbar muscular atrophy (SBMA)(Kennedy disease)	AR	Xq11–12	Androgen receptor	>38 (38–70)	9–36	transcription factor; nuclear receptor	1–2/100,000 male	spinal cord, brainstem	weakness, muscular atrophy, bulbar palsy	nucleus, cytoplasm

All the polyQ diseases are inherited in an autosomal dominant way, except for SBMA, which is X-linked.^23^ Although animal models of polyQ disease for understanding pathology in humans and exploring disease-modifying therapies are available, there is **neither a cure nor prevention for these diseases**, and only symptomatic treatments for polyQ diseases currently exist.^26^ The frequency of polyQ diseases is ∼1–10 cases per 100,000 people.^26,30^ HD and SCA3 have the highest occurrence worldwide.^31,32^ DRPLA predominantly takes place in Japan,^33^ while SBMA has been reported with a high frequency in Finland.^34,35^ Although each disease is considered rare, the polyQ diseases constitute the largest group of monogenic neurodegenerative disorders.^21^ The disease appearances are usually observed when the number of glutamines is beyond ∼35–45 (Table 1). However, for SCA6, the pathological threshold is ∼20 repeats, and for DRPLA and SCA3 it is ∼50–55 repeats (Table 1).^36^

### PolyQ Disease Pathogenesis and Molecular Mechanisms

PolyQ disorders exhibit certain common clinical and pathological features despite the fact that their single common genetic attribute is the pathogenic CAG repeat expansion and their affected genes are otherwise unrelated (Table 1).^38^ PolyQ diseases are all neurodegenerative disorders, with onset usually in midlife and slowly progressing phenotypes. These diseases demonstrate preferential degeneration of distinct cell types. The age of onset is inversely proportional to the CAG tract length, with this relationship being complex. Less common cases of childhood-onset disease occur in children with very long CAG repeats. Furthermore, the expanded repeat is unstable and may increase in length from one generation to the next. Expanded polyQ mutations are believed to cause cellular toxicity as a result of misfolded mutant proteins. All polyQ diseases except for SBMA are inherited in an autosomal dominant way, while SBMA is caused by a CAG repeat expansion in the X-linked androgen receptor gene, with the disease occurring only in men.

#### Normal Functions of Polyglutamine Disease Proteins

Huntington’s disease is caused by a polyQ expansion in huntingtin (HTT) protein, a nucleo-cytoplasmic protein, which has been shown to participate in axonal transport and in calcium signaling regulation. The androgen receptor (AR), the CAG–polyQ repeat expansion of which causes SBMA, is a nuclear receptor, best known for its roles in male reproductive system. Ataxin-1, the protein related to SCA1, is involved in RNA processing and transcriptional repression. Atrophin-1, the causal protein in DRPLA, is a transcriptional corepressor. Ataxin-3 is a ubiquitin editing enzyme and mediates the ubiquitinated proteins degradation. Ataxin-7 is a member of the STAGA acetyl-transferase coactivator complex and participates in transcription regulation.^23^

#### Misfolding and Aggregation of Mutant Polyglutamine Proteins

**Figure 3:**
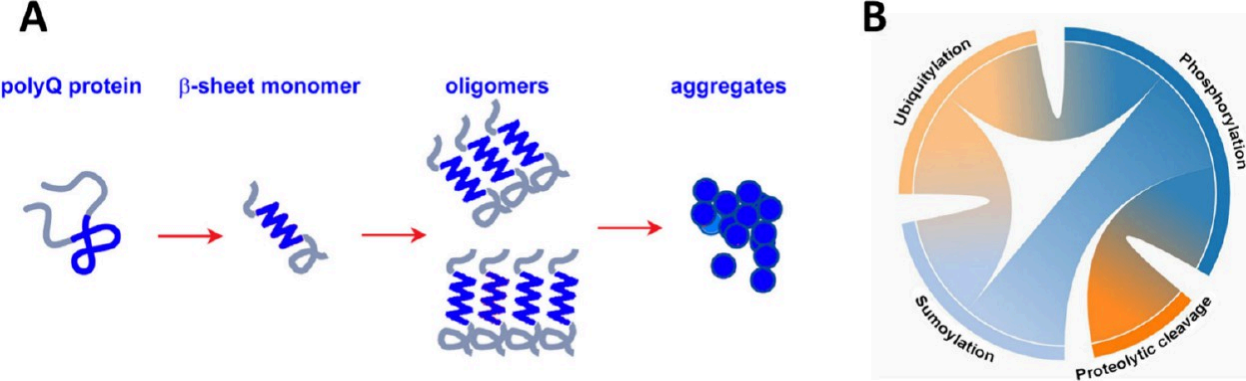
(A) Scheme of the aggregation pathway of the expanded polyglutamine (polyQ) proteins; (B) Post-translational modifications of the mutant polyglutamine proteins and their relationship.

The polyQ diseases are caused by an abnormal expansion of the polyQ tract in the disease-causing proteins. Proteins with an abnormally expanded polyQ stretch undergo a conformational transition to β-sheet rich structure, which assemble into insoluble aggregates with β-sheet rich amyloid fibrillar structures (Figure 3A) and accumulate as inclusion bodies in neurons, eventually leading to neurodegeneration. It has been thus suggested that polyQ diseases result from a toxic gain of function at the protein level, with a key pathological feature being the accumulation of aggregated polyQ proteins inside neurons nucleus and cytosol.^46,47^ However, while abnormal accumulation of the polyQ proteins such as inclusion bodies is one of the foremost pathological hallmarks commonly detected in the brains of the polyQ disease patients, the roles of aggregate/inclusion formation in disease pathogenesis have been controversial and remain one of the challenging problems in the field.

Indeed, aggregates and inclusions are usually found in affected areas of the brain as compared to the unaffected ones, and the late onset and progressive nature of polyQ diseases matches the slow process of protein aggregation. Some researchers have suggested that oligomeric aggregates such as protofibrils and microaggregates are the immediate cause of polyQ toxicity and that large aggregates are in fact cytoprotective.^31,48^ A variety of cellular proteins, including molecular chaperones, cytoskeletal proteins, transcriptional factors and proteasomes, have been detected into the inclusion bodies, suggesting detrimental effects on a wide range of essential cellular functions, which possibly contribute to neuronal dysfunction and eventual neuronal loss in various regions of the brain.^49−51^

#### Post-Translational Modifications of Mutant Polyglutamine Proteins

Although the mechanism of pathogenesis of polyQ diseases is still not fully characterized and understood, evidence suggests that post-translational modifications of the polyglutamine proteins are involved in their neurotoxicity and can significantly modulate it.^22,23,52,53^ Posttranslational modifications take place on amino acids out of the polyQ tract and frequently affect protein–protein interactions or function.

The pathogenesis of certain polyQ disorders including HD, SBMA, and SCA3, are likely related to **proteolytic cleavage** resulting in production of toxic polyQ-containing fragments.^54−57^ It is worth noting though that proteolytic cleavage is possibly not a critical step in all polyQ diseases pathogenesis. Indeed, for polyQ protein normally localized in the cytoplasm, such as htt, ataxin-3, or atrophin-1, proteolysis would facilitate their translocation into nucleus thus increasing their toxicities, while for proteins already localized in the nucleus, such as TATA-binding protein or ataxin-7, proteolytic cleavage may not play a role.

**Phosphorylation** can modify protein function, localization, and conformation by interfering with protein–protein interactions. It has been reported that in ataxin1, phosphorylation at serine 776 is dependent upon the length of the polyQ tract. Replacing this residue with an alanine thus preventing its phosphorylation considerably reduced cerebellar neuropathology in SCA1: polyQ ataxin-1 in which serine 776 is mutated to alanine displayed substantially reduced Purkinje cell degeneration.^58^ In another example, taking into account that expanded polyglutamine androgen receptor (AR) is phosphorylated by Akt, it has been demonstrated that substitution of the AR at two Akt consensus sites, serine 215 and serine 792 in SBMA models, with aspartate, which mimics phosphorylation, blocked ligand binding and lessened toxicity.^59^

**Acetylation**, the binding of an acetyl group with lysine residues in a protein, is a modification known to modulate protein–protein and protein–DNA interactions and control, typically increasing, protein stability. In SCA7, acetylation at lysine 257 elevates protein stability, and is suggested important for disease toxicity.^60^ In SBMA, acetylation at lysine 632/633 controls nuclear entry as well as the folding of androgen receptor protein.^61^

**Ubiquitination** is a highly specific biological process executed by a complex cascade of enzymes. It is essential for protein homeostasis, intricately involved in the regulation of protein quality control, operating to rapidly remove unwanted or damaged proteins.^62^ Certain neurodegenerative diseases including polyQ diseases are associated with the dysfunction of specific ubiquitin ligases.^63^ Recent studies implicate the ubiquitination machinery as a likely therapeutic target in polyQ diseases, since the ubiquitination system enzymes play a key role in targeting proteins for degradation both by the proteasome and autophagy.^64,65^

Small ubiquitin-like modifier (SUMO) proteins are known to bind to specific lysine residues in target proteins modifying their cellular localization, protein–protein interactions, and transcription factors activation.^66^**SUMOylation** refers to the SUMO moiety being attached to a lysine residue in the target protein. SUMO has been detected to colocalize with neuronal inclusion bodies in the brains of patients and cell models with HD, SCA3, and DRPLA thus suggesting that SUMO modification contributes to neurodegeneration in polyQ disease.^67,68^

Furthermore, certain post-translational modifications can be mutually linked to one another. For example, phosphorylation impacts the proteolytic cleavage of polyQ proteins; it can also affect SUMOylation and ubiquitylation.^53,69−71^ SUMOylation and ubiquitylation can compete for the same lysine residues, as suggested for htt, etc. (Figure 3B).^68^

#### Protein Degradation Pathways and Their Malfunction in PolyQ Diseases

Eukaryotic cells have two major systems to degrade toxic and misfolded proteins: the ubiquitin-proteasome system (UPS), and the autophagy–lysosomal pathway. The UPS is the route by which a cell degrades soluble, short-lived and misfolded proteins. Evidence has been provided that UPS function has been inhibited in polyQ diseases,^50^ implying that polyQ proteins cannot be degraded by the UPS system,^72,73^ so that misfolded polyQ-containing proteins must be degraded by autophagy. In brief, the process includes formation of a dual membrane, engulfing structures such as misfolded proteins, organelles and other substrates, forming autophagosomes, which further fuse with lysosomes so that lysosomal enzymes degrade the contents.^23^ The functional significance of the relation of UPS dysregulation and protein aggregation to disease pathophysiology remains unclear.

#### Abnormal Conformational Changes of Expanded PolyQ Proteins

In the initial studies exploring the molecular mechanisms of aggregate formation from the monomeric form of the expanded polyQ proteins it has been shown that synthetic polyQ peptides with a relatively short glutamine repeat of ∼15 form aggregates with β-sheet rich structures under certain conditions.^74^ PolyQ peptides with longer glutamine repeats gradually undergo a conformational change from a soluble random coil structure to insoluble amyloid-like fibrils with β-sheet structures.^75^ Intermediate structures such as oligomers and protofibrils are produced before amyloid formation, structurally similar to those of amyloid-β and α-synuclein formed in Alzheimer’s and Parkinson’s diseases, implying a shared pathogenic mechanism among the neurodegenerative diseases associated with protein aggregation.^76^ Therefore, the proposed aggregation cascade of the expanded polyQ proteins includes a conformational transition from the native conformer to the β-sheet rich structure in a monomeric state, which assembles into oligomers and insoluble aggregates with amyloid fibrillar structures, potentially leading to accumulation as intracellular inclusions (Figure 3).

A recent study examined the nucleation of pathologically expanded polyQ tracks and suggested that it involves segments of three glutamine (Q) residues at every other position. Molecular simulations suggested a pattern encoding a four-stranded steric zipper with interdigitated glutamine side chains.^77^ Once the initial zipper is formed, it engages naive polypeptides in a fashion characteristic of polymer crystals with intramolecular nuclei. By revealing the physical nature of the rate-limiting event for polyQ aggregation in cells, these findings elucidate the molecular etiology of polyQ diseases.^77^

#### Chromatin Structure and Epigenetic Regulation

An increased amount of data suggests that chromatin structure and epigenetic regulation are related to polyQ disease pathology. These diseases often exhibit abnormal transcriptional regulation. Epigenetic-related factors and chromatin structure are considered involved in genomic instability of CAG repeats. It has been concluded that disrupted chromatin regulation may be directly involved with the pathophysiology of polyQ diseases.^78^ The acetylation of histone by histone acetyl transferase plays an important role in the regulation of gene transcription.^79^ Data suggests that expanded polyglutamine proteins alter the histone acetylation and decrease gene expressions. Furthermore, growing data suggest a potential therapeutic role for drugs that target chromatin, such as histone deacetylase (HDAC) inhibitors, in various polyQ models.

#### Transcriptional Dysregulation

Transcriptional dysregulation is believed to be a common attribute of polyQ disorders. However, the precise causes of transcriptional alterations and how they relate to the observed phenotype remain unclear. Transcriptional impairment differs in different diseases, possibly reflecting the specific functions of the disease-causing proteins. Various aberrant interactions between expanded polyglutamine proteins and transcriptional factors and cofactors have been revealed, such as CREB-binding protein, p300/CBP-associated factor, p53, Sp1, TAFII130, PQBP-1, etc.^80^

#### Mitochondrial Dysfunction

Impairments of mitochondrial functions is a major feature of polyQ diseases resulting in cell death through activation of apoptotic cascades. The process of mitochondrial dysfunction in polyQ diseases is accompanied by enhanced free radical production and oxidative damage and abnormal energy metabolism.^81−83^ Furthermore, expanded polyQ proteins have been reported to impair axonal trafficking, which possibly results in abnormal mitochondrial distribution and function providing additional causal link between mitochondria and polyQ diseases.^84,85^ Based on multiple studies, it can be recognized that mitochondrial impairment is a common feature in the polyQ diseases pathogenesis.^31^

#### Excitotoxicity

A key role of excitotoxicity in neurodegenerative diseases is being explored and gaining acceptance, but the underlying mechanisms of its participation in neurodegeneration are still uncertain. Excessive activation of glutamate receptors by excitatory amino acids results in a number of deleterious consequences, such as impairment of calcium buffering, free radical generation, activation of the mitochondrial permeability, and secondary excitotoxicity.^86,87^ Mutant polyQ proteins disrupt calcium signaling pathways, leading to excessive calcium influx into neurons, impaired glutamate clearance, altered NMDA receptor function, activation of calcium-dependent proteases, excitotoxic neuronal damage, neuronal dysfunction and degeneration, dendritic spine loss, neuronal excitability alterations, and neuronal death, particularly in regions of the brain such as the cerebellum and brainstem.^86,87^

#### Genetic Basis of the PolyQ Diseases

The genetic abnormality underlying polyQ diseases involves the expansion of CAG trinucleotide repeats within the coding regions of specific genes. The CAG repeat encodes the amino acid glutamine. In healthy individuals, these repeats are present within a normal range. However, in individuals with polyQ diseases, there is an abnormal expansion of CAG repeats beyond the normal range (Table 1).^88,89^

- The expansion of CAG repeats occurs in a dynamic and unstable manner, leading to anticipation, a phenomenon where the severity of the disease tends to increase, and the age of onset tends to decrease in successive generations. This phenomenon is often observed in polyQ diseases, resulting in earlier onset and more severe symptoms in subsequent generations.^22,90^
- Eight of the polyQ diseases are inherited in an autosomal dominant manner, meaning that a single copy of the mutated gene is sufficient to cause the disease. Therefore, individuals who inherit the expanded CAG repeat from an affected parent have a 50% chance of developing the disease. Exception is SBMA, which is inherited in an X chromosome-linked recessive manner, depending upon male levels of circulating androgens; this aspect of SBMA pathogenesis leads to the disease occurring only in men.^22,91^
- Different polyQ diseases are associated with expansions in different genes. For example: Huntington’s disease is caused by an expansion of CAG repeats in the huntingtin (HTT) gene; spinocerebellar ataxias (SCAs) are caused by expansions in various genes, including ATXN1, ATXN2, ATXN3, CACNA1A, ATXN7; dentatorubral-pallidoluysian atrophy (DRPLA) is caused by expansions in the ATN1 gene; spinobulbar muscular atrophy (SBMA) is caused by expansions in the androgen receptor (AR) gene.^22,78^
- Somatic hypermutation involves a programmed process of mutation affecting the variable regions of immunoglobulin genes.^92^ It is a vital process in the adaptive immune system that allows B cells to generate a wide variety of antibodies, specifically immunoglobulins. Furthermore, somatic hypermutation can play a complex and multifaceted role in disease pathogenesis, with both beneficial and detrimental effects depending on the context. While somatic hypermutation itself is not the cause of polyQ diseases, the mechanisms by which CAG repeats expand somatically in affected individuals’ cells are of great interest. Understanding these mechanisms could potentially lead to therapeutic approaches that target the process of repeat expansion and thereby mitigate the progression of polyQ diseases.^37,93^

In brief, the genetic basis of polyQ diseases involves the expansion of CAG trinucleotide repeats within specific genes, leading to the production of proteins with pathologically long polyglutamine tracts, which contribute to neuronal dysfunction and neurodegeneration.

## Major PolyQ Diseases

### Huntington’s Disease

Huntington’s disease (HD) is a debilitating autosomal-dominant disease. Its prevalence is ∼3–7/100,000 persons (Table 1).^40^ Presentation begins in approximately the fourth-fifth decade of life, with chorea as the most common symptom. HD may also present with personality alterations, memory decline, and mood disturbance. Cognitive changes take place and culminate in dementia following onset of the movement disorder. Chorea may also develop into bradykinesia and rigidity late in the disease. The primary pathological hallmark is severe atrophy of the striatum, which is reduced to a fraction of its original size.^94^ HD is triggered by a CAG repeat expansion in the first exon of the huntingtin (htt) gene. Affected patients exhibit 36–250 repeats, compared to healthy individuals having 6–35 repeats.^38^ HD displays several features common to polyQ repeat diseases, including enhanced toxicity of a polyQ-containing fragment. Full-length polyQ-htt is being cleaved in the cytosol, generating toxic fragments predominantly translocated to the nucleus and forming nuclear inclusions over time,^95,96^ with larger repeats inducing increased inclusion formation.^97^ Transcriptional dysregulation and aberrant chromatin remodeling are central features in HD pathology.^98^

### Spinocerebellar Ataxias (SCAs)

**SCA1** is an autosomal-dominant disease characterized by progressive cerebellar ataxia. It accounts for ∼6% of cerebellar ataxias worldwide. SCA1 presents gradual loss of balance and coordination, compromised cognition, gaze palsy, peripheral neuropathy, and motor control symptoms^99^ – features common for spinocerebellar ataxias.^26^ The predominant pathological attributes are atrophy, gliosis, and severe loss of Purkinje cells in the cerebellum. Patients experience coordination difficulties, including dysarthria, dysphagia, and ophthalmoplegia.^100^ SCA1 is caused by a CAG repeat expansion in the coding region of the ataxin-1 (ATXN1) gene. This repeat is highly polymorphic, with 6–44 triplets in healthy individuals. Increased phosphorylation of polyQ-expanded ataxin-1 contributes to SCA1 pathology, so inhibition of these kinases may provide a therapeutic target.^101^

**SCA2** is also an autosomal-dominant, progressive ataxia that accounts for ∼13% of cerebellar ataxia cases. The main characteristic clinical feature of SCA2 is exceedingly slow saccade eye movements. Other symptoms include difficulties with coordinated movement and action tremor (uncommon with the other ataxias), myoclonus, and reduction in appetite. Occasionally, SCA2 patients present with parkinsonian characteristics, or have extensive motor neuron disease.^102,103^ SCA2 patients typically exhibit atrophy of the cerebellum and brainstem, with severe degeneration of cerebellar Purkinje cells and granule cells combined with neuron loss and gliosis. Degeneration of the substantia nigra, producing parkinsonism, and/or atrophy of the frontotemporal lobes may occur sometimes.^104,105^ SCA2 is caused by a CAG repeat expansion in the ataxin-2 (ATXN2) gene.^106^ Unlike the other polyQ diseases, SCA2 patients exhibit mainly cytoplasmic, rather than nuclear, inclusions.^107^

**SCA3**, a.k.a. Machado–Joseph disease, is the most common autosomal-dominant cerebellar ataxia worldwide. SCA3 typically show symptoms in young adults or in middle age with slowly progressive and highly variable syndromes attributed to four clinical subtypes.^108,109^ Each of them has a core presentation of cerebellar ataxia, dysphagia, dystonia, pyramidal signs, progressive external ophthalmoplegia, and muscle atrophy, as well as weight loss and restless-legs syndrome.^108,109^ SCA3 is unique among other SCAs with neuron loss being often mild in Purkinje cells of the cerebellum.^110,111^ SCA3 is caused by a CAG repeat expansion near the 3′ end of the coding region of the ataxin-3 (ATXN3) gene.^112^ Alleles in healthy individuals range from 12 to 40 CAG repeats, while diseased ones carry 55–84 CAG repeats.^109,113^

**SCA6** is an autosomal-dominant, slowly progressive cerebellar ataxia. The average age of onset is in the fifth decade of life, with most patients presenting gait/upper-limb incoordination, intention tremor, dysarthria, dysphagia, and diplopia. Vertical nystagmus, horizontal gaze-evoked nystagmus, and difficulty fixating on moving objects are common clinical features. Neuropathology examination shows distinct cerebellar atrophy with severe loss of Purkinje cells and moderate loss of cerebellar granule cells.^114,115^ SCA6 is caused by a CAG repeat expansion in exon 47 of the CACNA1A gene.^114,115^ Pathogenic alleles range from 19 to 33 CAG repeats, while healthy individuals possess alleles with 4–18 CAG repeats.^116,117^ CAG repeat size differences are small in comparison to other polyQ repeat diseases, and somatic instability is minimal.^118^

**SCA7** is an autosomal-dominant, rapidly progressive cerebellar ataxia associated with visual impairment. SCA7 shows a wide geographic distribution, and a prevalence of ∼1/500,000.^119,120^ Patients display pronounced dysarthria and can develop visual impairment due to a cone–rod dystrophy form of retinal degeneration, ultimately leading to blindness.^121,122^ Neurodegeneration and reactive gliosis occur in the cerebellar cortex, dentate nucleus, inferior olive, pontine nuclei, and occasionally the basal ganglia. Cerebellar tissue from SCA7 patients exhibits extensive loss of cerebellar Purkinje cells.^123,124^ As with other polyQ diseases, nuclear inclusions are common in vulnerable populations.^125,126^ SCA7 is caused by a CAG repeat expansion at the 5′ end of the coding region of the ataxin-7 gene.^127,128^ While healthy individuals possess alleles ranging in size from 7 to 35 CAGs, diseased expanded SCA7 CAG repeats are among the most unstable of all coding repeat expansions, with patients having 37 to >300 repeats.^128^

**SCA17** is an extremely rare dominantly inherited cerebellar ataxia, with <100 families reported.^129^ Ataxia and psychiatric abnormalities are common at the beginning, later accompanied by chorea and dystonia, dementia, pyramidal signs, rigidity, and, rarely, parkinsonism.^130,131^ Neuropathology involves atrophy of the cortex, striatum, and cerebellum, with neuron loss in the striatum and cerebellar Purkinje cell layer.^131^ SCA17 is caused by a CAG repeat expansion in the TATA-binding protein gene on chromosome 6q27.^131,132^ Normal alleles contain 25–48 CAG repeats, while those in diseased individuals contain 43–66 CAG repeats. Age of onset is variable, from early childhood to middle age, and SCA17 ancestries exhibit anticipation.^133^ Patients also display nuclear inclusions.^134^

(Another spinocerebellar ataxia, SCA12, is also caused by a CAG repeat expansion, however it is not transcribed to polyglutamine–the repeat expansion is located in a noncoding region of a gene, so it is not considered a polyQ (polyglutamine) disease.^135,136^)

### Dentatorubral-Pallidoluysian Atrophy

Dentatorubral-pallidoluysian atrophy (DRPLA) is a very rare, dominantly inherited disorder reported in all populations, but most often in the Japanese population.^137^ Neuropathology is wide-ranging and includes degeneration of the dentatorubal and pallidoluysian circuits. Most DRPLA patients display progressive cerebellar ataxia, choreoatheosis, epilepsy, and dementia, while juvenile-onset cases include myoclonus and mental retardation.^138,139^ DRPLA is caused by an unstable CAG repeat in the atrophin-1 gene coding region. Normal alleles in the atrophin-1 gene contain 8–25 CAG repeats, while patient expansions range in 49–88 CAGs.^139,140^

### Spinal and Bulbar Muscular Atrophy

Spinal and bulbar muscular atrophy (SBMA) (a.k.a. Kennedy disease) is the only polyQ disease inherited in a sex-limited pattern, having a prevalence of ∼1 per 300,000 males. It is characterized by late-onset, progressive degeneration of lower motor neurons of the spinal cord and in the bulbar region of the brain stem,^141,142^ as well as sensory neurons in the dorsal root ganglia.^143^ The disease is caused by a polymorphic CAG repeat expansion in the first exon of the AR gene coding region. Affected patients exhibit 37–70 repeats, while unaffected ones have 5–34 repeats,^27^ and, similarly to other polyQ diseases, there is a correlation between disease repeat length and disease severity.^144,145^ There is no treatment available yet to alter the course of SBMA.

## Therapeutic Strategies

Treatment strategies for polyQ diseases primarily focus on managing symptoms, slowing disease progression, and improving quality of life.^26^ While there is currently no cure for these conditions, ongoing research aims to develop therapies that target the underlying mechanisms of polyQ diseases. Overall, a multidisciplinary approach involving collaboration between researchers, clinicians, and patients is crucial for advancing treatment strategies for polyQ diseases and improving outcomes for affected individuals.

- **Protein degradation enhancers**: Therapies that enhance the clearance of misfolded or aggregated proteins, such as autophagy enhancers and ubiquitin-proteasome system activators, are being investigated as potential treatments for polyQ diseases.^91,146^
- **Gene silencing therapies**: RNA interference (RNAi) and antisense oligonucleotide (ASO) therapies aim to reduce the expression of the mutated gene responsible for producing the abnormal polyglutamine protein. These treatments target the mRNA (mRNA) to prevent the production of the toxic protein, potentially slowing disease progression.^147−149^
- **Small molecule therapies** are being developed to target specific pathways involved in polyQ diseases, such as protein aggregation (i.e., to act as binding competitors to block the assembly between polyQ protein monomers), oxidative stress, and mitochondrial dysfunction. These molecules may help modulate disease mechanisms and alleviate symptoms.^150−153^
- **Protein misfolding and aggregation prevention by overexpression of endogenous chaperones:** Protein misfolding and aggregation are prevented by the machinery of molecular chaperones. Some chaperones such as the members of the Hsp70 family are known to modulate polyQ aggregation and suppress its toxicity.^154−158^
- **Neuroprotective compounds**, including antioxidants, anti-inflammatory agents, and neurotrophic factors, are being explored for their potential to protect neurons from degeneration and improve neuronal function in polyQ diseases.^159,160^
- **Stem cell-based therapies**, including cell replacement therapy and stem cell-derived exosome therapies, are being investigated for their potential to replace damaged cells, promote tissue repair, and modulate the microenvironment in the brain affected by polyQ diseases.^26,161−163^
- Emerging **gene editing technologies**, such as CRISPR-Cas9, hold promise for correcting the genetic mutations underlying polyQ diseases. However, challenges related to specificity, efficiency, and safety need to be addressed before these approaches can be applied clinically.^45,164,165^
- **Symptomatic treatments** aim to alleviate specific symptoms associated with polyQ diseases, such as movement disorders, cognitive impairment, psychiatric symptoms, and dysphagia. These treatments may include medications, physical therapy, occupational therapy, speech therapy, and assistive devices.^26,166−168^
- **Combination therapies** involving multiple treatment modalities, such as gene silencing with neuroprotective agents or stem cell transplantation with symptomatic treatments, may offer synergistic benefits and enhance overall therapeutic efficacy.^162,169−172^
- **Precision medicine approaches** aim to tailor treatments based on individual genetic profiles, disease characteristics, and response to therapy. Personalized treatment strategies may optimize therapeutic outcomes and minimize adverse effects.^173−176^

A list of representative drugs explored in pharmacological treatments of polyQ diseases is provided in Table 2, along with their suggested mechanism and targeted diseases. Huntington’s disease is currently the only polyQ disease that has US FDA approved treatments. Three drugs, Xenazine (tetrabenazine),^177^ Austedo (deutetrabenazine) both immediate and extended release,^178−180^ and Ingrezza (valbenazine)^181^ are approved for the treatment of the movement disorder chorea, caused by Huntington’s disease. Xenazine received approval in 2008, followed by Austedo in 2017, along with Austedo XR and Ingrezza last year in 2023.

**Table 2:** Drugs explored in pharmacological treatments of polyQ diseases^146,166,182−187^

**Drug**	**Brand name**	**CAS RN**	**Drug class**	**Mechanism**	**Treats**
Acetazolamide		59–66–5	Carbonic anhydrase inhibitor	Autophagy modulator; lower blood pH and carbonic anhydrase inhibitor	SCA1; SCA6
Amantadine		768–94–5	Antiglutamatergic; Adamantane	Noncompetitive *N*-methyl-d-aspartate (NMDA) receptor antagonist; Increases dopamine release	HD; SCA7
4-aminopyridine (4-AP)	Fampridine	504–24–5	Potassium channel blocker	Ameliorate motor coordination deficiency	SCA1; SCA6
Aripiprazole	Abilify	129722–12–9	Atypical antipsychotic		HD
B vitamins		12001–76–2	Vitamins		SCA2
Baclofen		1134–47–0	Skeletal muscle relaxant	GABA receptor agonist	SCA1; antispasmodic agent
Buspirone		36505–84–7	Snxiolytic agent; serotonin 5-HT1A receptor agonist	Autophagy modulator; Prevents dopamine reuptake	HD; SCA7
Butyrophenone		495–40–9	Major tranquilizer		HD
Carbamazepine		298–46–4	Mood stabilizer	Blockade of voltage-gated sodium ion channels	SCA17; anticonvulsant
Chromomycin		74913–06–7	Antitumor antibiotic		HD
Citalopram		59729–33–8	Antidepressant (Selective serotonin reuptake inhibitor (SSRI))	Inhibit the reuptake of 5-HT into the presynaptic nerve terminal	SCA3
Clonazepam		1622–61–3	Benzodiazepine	Autophagy modulator; Modulation of GABA function in the brain	DRPLA
Chlordiazepoxide		58–25–3	Benzodiazepine	Increased binding of the inhibitory neurotransmitter	DRPLA
Chlorzoxazone		95–25–0	1,3-Benzoxazole	Potassium channel modulator; improve cerebellar electrophysiology	SCA2
Clozapine		5786–21–0	Atypical antipsychotic	Binds to the dopamine D4 receptor with a higher affinity than the dopamine D2 receptor	HD
Coenzyme Q10		303–98–0	Vitamin-like		HD
Curcumin		458–37–7	Curcuminoids	Aggregation inhibitor	HD
Deutetrabenazine	Austedo	1392826–25–3	VMAT2 inhibitor	Depletes central monoamines by reversibly inhibiting VMAT2	chorea
Donepezil		120014–06–4	Cholinesterase inhibitor		HD
Dutasteride		164656–23–9	5-Alpha reductase inhibitors	Autophagy modulator; Decreases DHT production	SBMA
EGCG		989–51–5	Catechin gallates		HD
Ethyl-eicosapentaenoic acid (EPA)	Vascepa	86227–47–6	Omega-3 Fatty Acid		HD
Fasinumab		1190239–42–9	Monoclonal antibody	Autophagy modulator; Inhibiting the binding of NGF	SCA3
Fluoxetine		54910–89–3	Antidepressant (SSRI)	Inhibit the reuptake of 5-HT into the presynaptic nerve terminal	HD
Guanabenz		5051–62–7	Antihypertensive; alpha-2 adrenergic receptor agonist	Antiaggregation	HD
Haloperidol	Haldol	52–86–8	Antipsychotic		HD; chorea
Interferon β		145258–61–3	Immunomodulator		SCA7
Lamotrigine		84057–84–1	Mood stabilizer	Blockade of voltage-gated sodium ion channels	SCA17
Levodopa		59–92–7	Dopamine agonist	Alleviate rigidity/bradykinesia	SCA2
Liraglutide		204656–20–2	Incretin mimetic	Autophagy modulator; AMPK activation	HD
Lisuride	Dopergin	18016–80–3	Ergoline monoaminergic		SCA2
Lithium		7439–93–2	Antimanic	Autophagy modulator; mTORC1 inhibition	HD; SCA2
Memantine		19982–08–2	central nervous system agent	*N*-Methyl-d-Aspartic Acid Receptor Antagonist	HD
Methylene blue		61–73–4	Phenothiazine	Aggregation inhibitor	HD
Mirtazapine		85650–52–8	Tetracyclic antidepressant		HD
Mithramycin		18378–89–7	Anthracycline antibiotic		HD
Myricetin		529–44–2	Flavonoid		HD
Naphthyridine-azaquinolone		500722–22–5	Naphthyridine		HD
Neferine		2292–16–2	Bisbenzylisoquinoline alkaloid	Autophagy modulator; AMPK activation	HD
Olanzapine		132539–06–1	Atypical antipsychotic	Inhibits dopamine receptors, serotonin receptors, histamine receptors as well as a1-adrenergic and muscarinic receptors	HD
Perampanel		380917–97–5	Anticonvulsant		DRPLA
Phenothiazine		92–84–2	Heterocyclic antipsychotic		HD
Pimozide		2062–78–4	Antipsychotic	Neuroleptic drug selectively blocks dopamine receptor D2 (DRD2)	HD
Piperine		94–62–2	NF-kappaB inhibitor		SCA17
Piracetam		7491–74–9	Nootropic	Autophagy modulator; Producing a lowering of cerebral artery tonus	DRPLA
Pridopidine		346688–38–8	Dopaminergic stabilizer	Dopamine D2 receptor (D2R) antagonist	HD
Propranolol		525–66–6	Beta blocker		HD
Quetiapine		111974–69–7	Atypical antipsychotic		HD
Rapamycin		53123–88–9	Macrocyclic immunosuppressant	Autophagy modulator; mTORC1 inhibition	HD
Reserpine		50–55–5	Auwolfia alkaloid		HD
Rilmenidine		54187–04–1	Centrally active antihypertensive	Autophagy modulator; AMPK activation	HD
Riluzole		1744–22–5	Antiglutamatergic	Autophagy modulator; Inhibits the release of glutamic acid from cultured neurons	SCA2; SCA6; SCA7
Risperidone	Risperdal	106266–06–2	Atypical antipsychotic	Selectively inhibiting serotonin and dopamine-D2 receptors	HD
Rivastigmine		123441–03–2	Cholinesterase inhibitor		HD
Salubrinal		405060–95–9	eIF2α dephosphorylation inhibitor		HD
Sertraline		79617–96–2	Antidepressant (SSRI)	Inhibit the reuptake of 5-HT into the presynaptic nerve terminal	HD
Tanezumab		880266–57–9	Monoclonal antibody	Autophagy modulator; Inhibiting the binding of NGF	SCA3
Tetrabenazine	Xenazine	58–46–8	VMAT2 inhibitor	Depletes central monoamines by reversibly inhibiting VMAT2	HD; chorea
Topiramate		97240–79–4	Second-generation antiepileptic	Autophagy modulator; Control brain activity	SCA17
Trehalose		99–20–7	Disaccharide	Autophagy modulator; mTORC1 inhibition	HD
Valbenazine	Ingrezza	1025504–45–3	VMAT2 inhibitor	Monoamine-depleting agent	tardive dyskinesia, chorea
Valproate, sodium salt		1069–66–5	Mood stabilizer	Autophagy modulator; Mediated through effects on the function of brain	DRPLA; SCA17; anticonvulsant
Varenicline		249296–44–4	Nicotinic agonist	Partial agonist at α4β2 neuronal nicotinic acetylcholine receptor; improve axial symptoms and rapid alternating movements	SCA3
Venlafaxine		93413–69–5	Selective serotonin and norepinephrine reuptake inhibitor (SNRI)		HD
Zinc sulfate		7733–02–0	Mineral		SCA2
Ziprasidone	Geodon	146939–27–7	Atypical antipsychotic	Antagonist at D2, 5HT2A, and 5HT1D receptors, and an agonist at the 5HT1A receptor; inhibits synaptic reuptake of serotonin and norepinephrine	HD

Additional assistive remedies and approaches that can help manage symptoms and improve quality of life of the polyQ diseases patients include:

- **Physical therapy** can help maintain mobility and independence by improving muscle strength, flexibility, balance, and coordination. Occupational therapy can also assist in adapting daily activities to the individual’s abilities.
- **Speech therapy** may be beneficial for individuals experiencing speech and swallowing difficulties, common in some polyQ diseases.
- **Assistive devices** such as walkers, wheelchairs, communication aids, and other assistive technologies can enhance mobility and communication abilities.
- **Nutritional support** is essential for overall health. Some individuals with polyQ diseases may require modified diets or feeding tubes to address swallowing difficulties or weight loss.
- **Genetic counseling** can provide information and support for individuals and families affected by polyQ diseases, including guidance on family planning and genetic testing.
- **Support groups and counseling** can offer emotional support, practical advice, and an opportunity to connect with others facing similar challenges.
- Participation in **clinical trials** can provide access to experimental treatments and contribute to the advancement of research into polyQ diseases.
- Adopting a **healthy lifestyle**, including regular exercise, adequate rest, stress management techniques, and social engagement, can help improve overall well-being and possibly slow disease progression.

### Drug Delivery Systems

Traditional drug delivery vectors exhibit physicochemical characteristics that limit their ability to pass through biological barriers, in particular the blood-brain barrier, to reach the brain, which is the main target of the polyQ disease therapeutics. To improve the brain bioavailability of therapeutic active agents, new formulations based on nanocarriers have emerged.

Essential requirements for such nanocarriers are high stability and specificity, suitable tissue distribution, satisfactory cell penetration and efficient cytoplasmic or nuclear delivery.^182^ Due to the advantage of their size, nanoscale systems have been shown to be efficient drug delivery systems and may be useful for encapsulating drugs, enabling more precise targeting with a controlled release. Their use may address some of the most pressing challenges in drug delivery, such as solubilizing poorly water-soluble drugs, protecting labile drugs from degradation, and delivering drugs selectively to disease sites. These nanosized structures penetrate tissue, facilitate efficient uptake of the drug by cells, enable successful drug delivery, and ensure activity at the targeted location. The uptake of nanostructures by cells is much higher than that of large particles.^188,189^ Modifying or functionalizing nanoparticles to deliver drugs through the blood-brain barrier for targeting maladies of the central nervous system has been one superb outcome of medical nanotechnology.^190^ Since a wide variety of nanoparticles made of biological and/or synthetic materials are available, selecting suitable particles relies mainly on the type of therapeutic molecule to be delivered.

**Figure 4:**
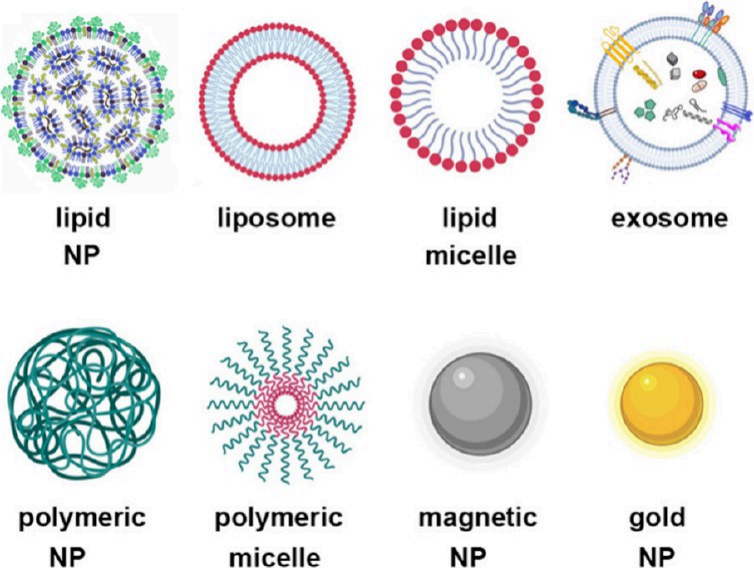
Schematic presentation of nanoparticulate drug delivery systems used in polyQ disease treatment.

Nanoparticular carrier systems designed for brain delivery of therapeutic drugs are composed of different materials, including mainly polymers, lipids, metals, or a combination of these materials (Figure 4). In addition, nanoparticle (NP) formulations must provide high biocompatibility, biodegradability, low toxicity, and protein-mediated opsonization. Moreover, clearance by the reticuloendothelial system is an obstacle for NPs to overcome.^191−195^

**Lipid-based NPs** are widely used as carriers in drug and gene delivery.^193^ Various nanostructures have been utilized including solid lipid NPs, liposomes, and micelles (Figure 4). For instance, stable nucleic acid lipid particles, incorporating a short peptide derived from rabies virus glycoprotein and encapsulating small interfering siRNAs, has been engineered to target mutant ataxin-3 in mouse models of SCA3 and reported successful in ameliorating motor behavior and neuropathological alterations.^196^ Solid lipid NPs composed of hydrogenated soya phosphatidylcholine 3-nitropropionic acid were used to deliver intranasally the neuroprotector rosmarinic acid to the brain of rats for effective management in Huntington’s disease.^197^ Furthermore, hybrid polymer/lipid NP systems comprising poly(lactic-*co*-glycolic acid) and solid lipid (Witepsol E85) were functionalized with a peptide-binding transferrin receptor to enhance their capacity to cross the blood-brain barrier to target human brain endothelial cells and deliver siRNAs.^198^

**Polymeric NP** are another successful pharmaceutical nanocarrier.^192^ Hydrophobic polymers such as poly(l-lysine), polyethylenimine, poly(lactic acid), poly(lactic-*co*-glycolic acid), and poly(ε-caprolactone), as well as hydrophilic polymers including chitosan, alginate, gelatin, and hyaluronic acid, have been commonly used.^182^ These polymers, alone or in combination, can entrap biomolecules of interest for molecular therapies. For example, brain distribution of aripiprazole, a small molecule drug reducing the levels of mutant ataxin-3 protein,^196,199^ was expedited when encapsulated into poly(ε-caprolactone) NPs and administered intranasally to rats.^200^ Poly(ε-caprolactone)/ Pluronic F-68 NPs have been reported effective in mediating the delivery of the antioxidant curcumin to neural-like cells.^201^ NPs based on natural polymers such as chitosan and cyclodextrins have been found effective delivery systems because of their ability to cross the blood–brain barrier.^202,203^ Self-assembling modified β-cyclodextrin nanoparticles were applied as neuronal siRNA delivery vectors and reported successful in alleviating motor deficits in mouse model of Huntington’s disease.^204^

**Metallic NPs** are also efficient pharmaceutical nanocarriers because of their unique physiochemical properties. Aggregation of polyQ-containing mutant huntingtin has been hindered in neuronal cells in Huntington’s disease mouse brains by using an Fe2O3 polyacrylate-coated and covalently conjugated poly(trehalose) nanocarrier system.^205^ A similar metallic NP-based approach may be scaled to treat polyQ SCAs.

### Biomarkers

Biomarkers for polyQ diseases play a crucial role in diagnosis, prognosis, and monitoring disease progression.^39,206−208^

- **Mutant protein aggregates**: In polyQ diseases, mutant proteins tend to aggregate within affected neurons. Detection of these aggregates, either through imaging techniques like PET scans or in post-mortem brain tissue, can serve as a biomarker.^209,210^
- **Cerebrospinal fluid (CSF) biomarkers**: Analysis of CSF can reveal changes in levels of certain proteins associated with neurodegeneration, such as tau, neurofilament light chain (NfL), and specific fragments of huntingtin protein in Huntington’s disease. In another example, cocaine- and amphetamine-regulated transcript (CART) is reported elevated in CSF of HD patients, possibly due to the pathogenic lesions in the hypothalamus.^211^ The oxidative stress marker F2-isoprostane is also increased in CSF from HD patients.^212^ CSF levels of homovanillic acid are declined in CSF samples from HD, SCA1, and SCA3 patients, as a result of the altered dopamine metabolism.^213,214^ Lactate/pyruvate ratio is elevated in CSF of HD and SCA3 patients.^215^
- **Peripheral biomarkers**: Blood-based biomarkers are of particular interest due to their noninvasive nature. These may include levels of mutant protein fragments, microRNAs, or other molecular signatures indicative of disease status. Given the implication of mitochondrial damage in the pathogenesis of HD and other polyQ diseases, causes of oxidative stress are plausible markers to monitor disease progression. For example, the serum level of 8-hydroxydeoxyguanosine (8-OHdG), an indicator of oxidative damage to DNA, has been found increased in HD patients.^216^ The amounts of mitochondrial DNA from leukocytes, which is another marker of oxidative stress, has declined in the blood from patients with polyglutamine diseases.^217^
- **Neuroimaging markers**: Techniques like MRI can detect structural changes in the brain associated with polyQ diseases, such as cortical atrophy, white matter abnormalities, or changes in specific brain regions affected by the disease.^218,219^
- **Electrophysiological markers**: Changes in electrical activity in the brain or peripheral nerves may serve as biomarkers. For example, abnormalities in electroencephalography (EEG) or nerve conduction studies can provide insights into disease progression.^220,221^
- **Biomarkers of oxidative stress and inflammation**: PolyQ diseases are associated with increased oxidative stress and neuroinflammation. Biomarkers related to these processes, such as markers of lipid peroxidation, cytokine levels, or markers of glial activation, may be indicative of disease severity.^222−224^
- **Metabolic biomarkers**: Metabolic dysregulation is increasingly recognized as a feature of neurodegenerative diseases. Biomarkers related to energy metabolism, mitochondrial function, or metabolite levels in the brain or peripheral tissues could provide insights into disease mechanisms and progression.^225,226^
- **Genetic modifiers**: Variants in other genes can modulate the age of onset, severity, or progression of polyQ diseases. Identifying genetic modifiers through genome-wide association studies (GWAS) or whole-genome sequencing could help stratify patients based on disease risk and prognosis.^37,227,228^

A combination of biomarkers, along with clinical assessment, can aid in the diagnosis, prognosis, and monitoring of polyQ diseases, as well as in the development and evaluation of potential therapies.

## Landscape of PolyQ Disease Research

**Figure 5:**
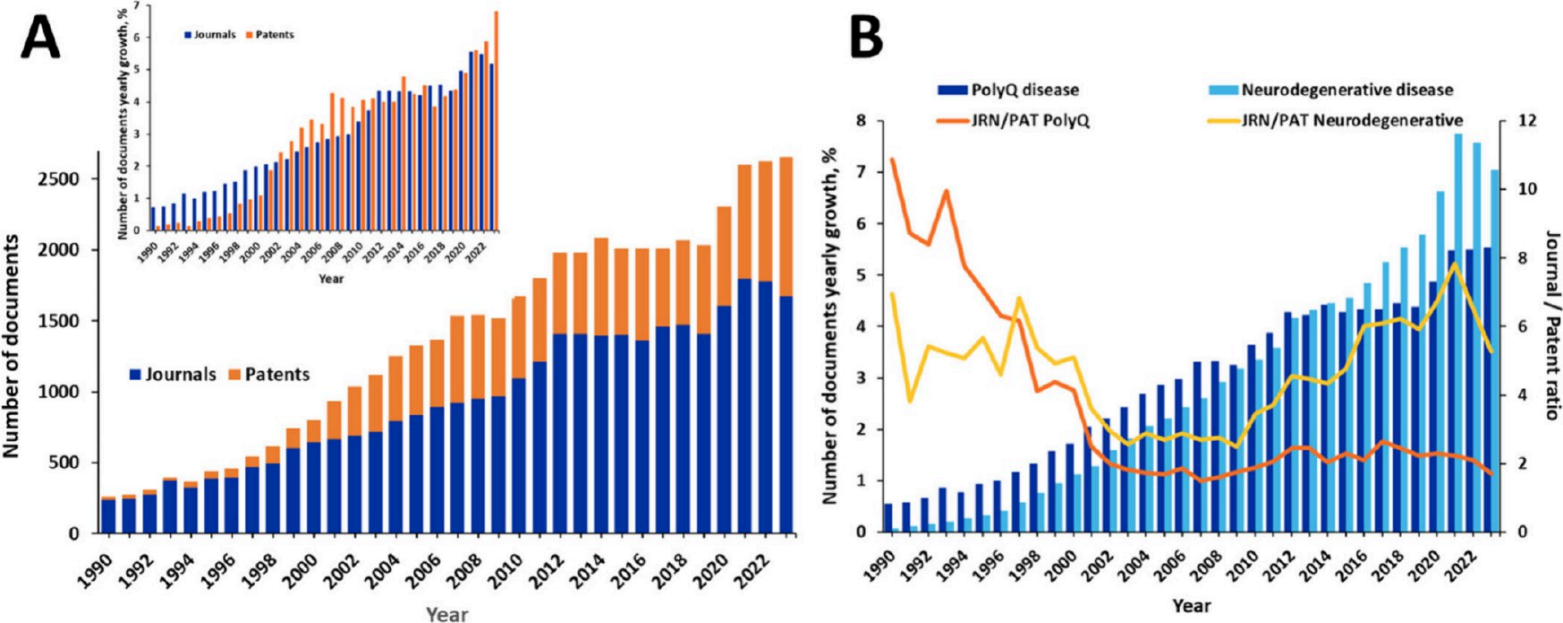
(A) Yearly trend of the number of documents (journal articles and patents) in the CAS Content Collection related to polyQ diseases, including Huntington’s disease, spinocerebellar ataxias, SBMA and DRPLA; Inset: relative growth in the journal and patent publications; (B) comparison between relative growth in the number of documents related to polyQ diseases (dark blue bars) and all neurodegenerative diseases (light blue bars); orange and yellow lines compare the journal/patent ratio for the class of polyQ disease and all neurodegenerative diseases, respectively.

The CAS Content Collection^20^ is the largest human-compiled collection of published scientific information. It represents a valuable resource to access and keep up to date on the scientific literature all over the world, across disciplines, including chemistry, biomedical sciences, engineering, materials science, agricultural science, and many more. This allows quantitative analysis of global research publications across various parameters including time, geography, scientific area, medical application, disease, and chemical composition. Currently, there are over 50,000 scientific publications (mainly journal articles and patents) in the CAS Content Collection related to polyQ diseases, including Huntington’s disease, spinocerebellar ataxias, SBMA and DRPLA. There has been a steady growth of these documents over the last three decades, with an >25% increase in the last three years (Figure 5A). Noteworthy, while in the earlier years, scientific journal publications notably dominated (journal/patent ratio >4–5), after around the year 2002 the number of patents exhibited significant growth (journal/patent ratio ∼2) (Figures 5A inset, 5B), correlating with the initial accumulation of scientific knowledge and its subsequent transfer into patentable applications.

In Figure 5B, the relative growth in the number of documents and the journal/patent ratio for the polyQ disease are compared to those for the general class of neurodegenerative disease. While the growth in the polyQ documents was faster in the previous years, it considerably lags behind that of the general neurodegenerative disease class in the recent decade. This might be a result of the lack of major breakthroughs in the polyQ disease research in recent years. Indeed, while there is a significant research progress in other neurogenerative diseases such as, for example, Alzheimer’s disease, which resulted in the recently approved by the US FDA drug lecanemab,^229^ and more drugs and treatments on the horizon,^230,231^ as well as for Parkinson’s disease, for which the noninvasive ultrasound treatment Exablate Neuro has been recently approved,^232,233^ there is still no cure for any of the polyQ diseases. The higher journal/patent ratio for the general field of neurodegenerative disease as compared to the polyQ disease (Figure 5B) also points out more active research in the former.

**Figure 6:**
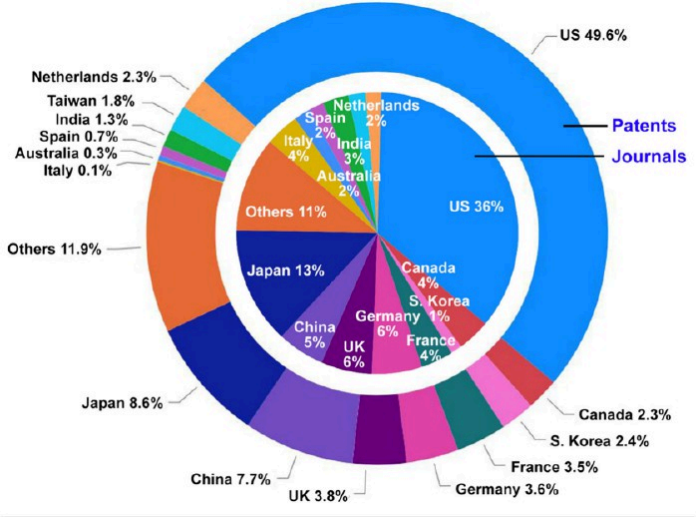
Top countries/regions with respect to the percentage of polyQ disease-related journal articles (inner pie chart) and patents (outer donut chart) in the CAS Content Collection.

**Figure 7:**
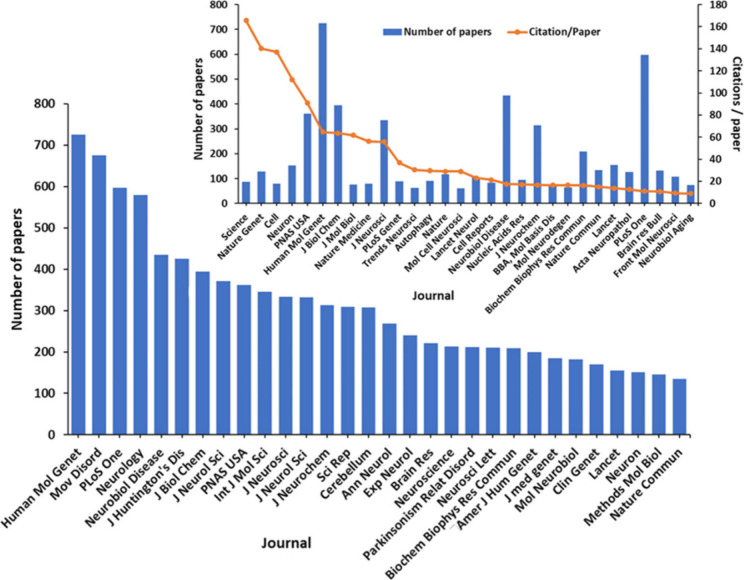
Leading scientific journals in the field of polyQ research based on journal publication data from the CAS Content Collection for the period 2003–2023. Blue bars represent number of journal publications while the orange line in the inset represents the average number of citations per publication.

The United States, Japan, China, the United Kingdom, Germany, France, Canada, and South Korea are the leaders with respect to the number of published journal articles and patents related to the polyQ disease research, with ∼1/3 of the journal articles and ~half of the patents coming from the United States (Figure 6). The journals *Human Molecular Genetics*, *Movements Disorders, PLoS One, and Neurology* have published the highest number of articles related to general polyQ research (Figure 7), while *Science, Nature Genetics,* and *Cell* have received the highest average number of citations per article, an indicator of the impact of journal publications (Figure 7, Inset).

**Figure 8:**
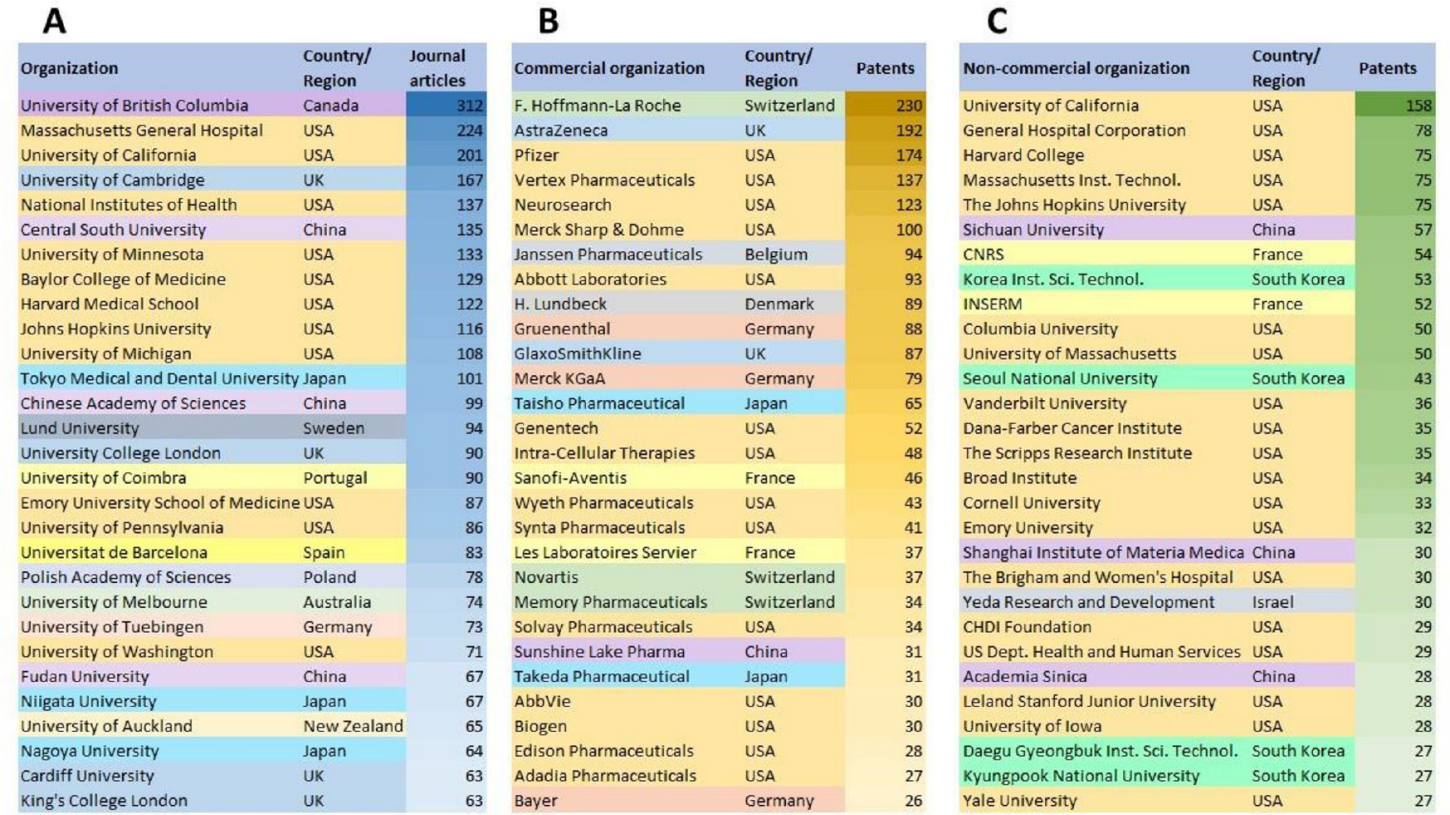
Leading organizations in the field of polyQ diseases in terms of number of published journal articles (A) and patents by (B) commercial and (C) noncommercial organizations.

The University of British Columbia, Massachusetts General Hospital, the University of California, and the University of Cambridge have the largest number of published articles in scientific journals (Figure 8A). Patenting activity is dominated by corporate players as compared to academics (Figure 8B,C). F. Hoffmann-La Roche, AstraZeneca, Pfizer, and Vertex Pharmaceuticals have the highest number of patent applications among the companies (Figure 8B), while the University of California, General Hospital Corporation, and Harvard College lead among the noncommercial organizations (Figure 8C).

**Figure 9:**
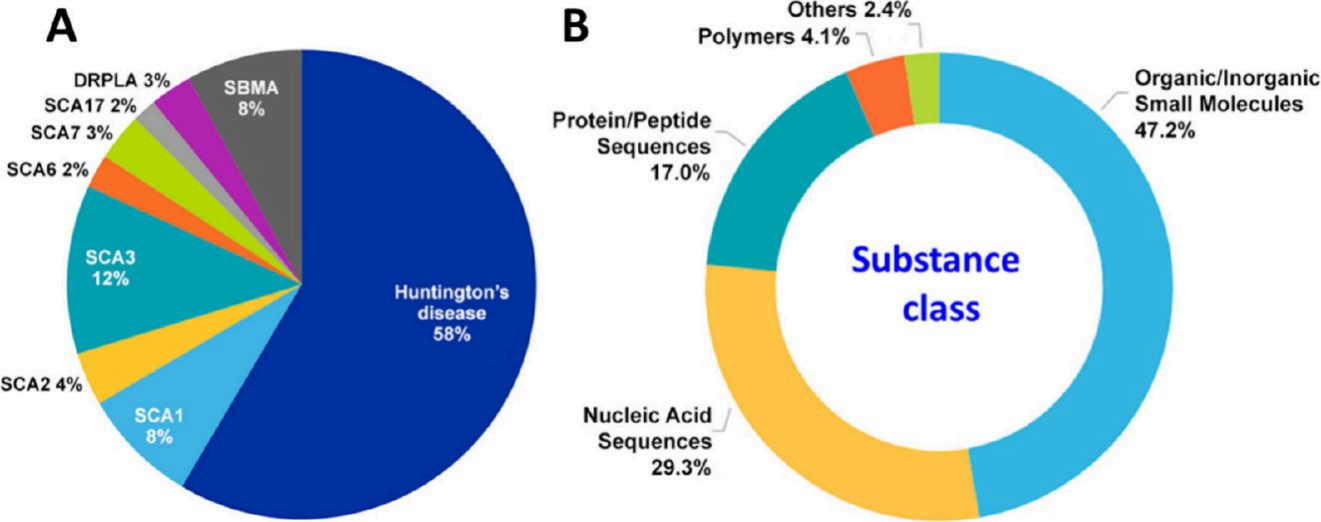
(A) Distribution of the number of documents in CAS Content Collection related to the various polyQ diseases; (B) Distribution of the major substance classes between the documents related to the polyQ diseases.

More than a half of the documents related to polyQ diseases in the CAS Content Collection are associated with Huntington’s disease, followed by SCA3, SCA1, and SBMA (Figure 9A), which roughly correlates with the worldwide prevalence of these diseases (Table 1). With respect to the substance classes, the largest part belongs to the organic and inorganic small molecules (Figure 9B).

**Figure 10:**
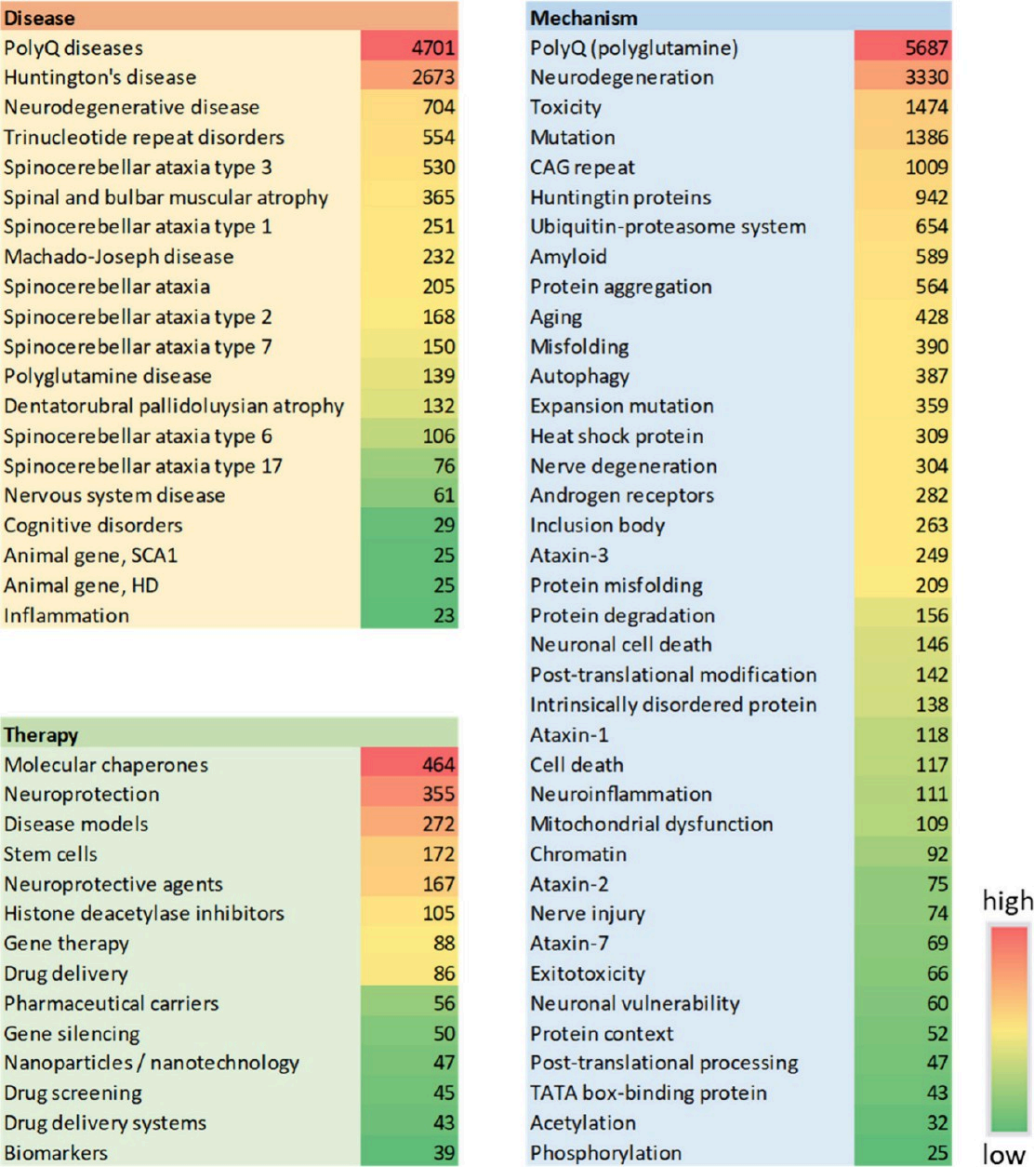
PolyQ disease-related concepts explored in the CAS Content Collection, associated with disease, therapy and disease mechanism.

We further explored distribution of the various polyQ disease-related concepts in the published documents (journals and patents) (Figure 10).

**Molecular chaperones** rang at the top of the therapeutic strategies concepts. Molecular chaperones play a key role in maintaining cellular proteostasis by facilitating the correct folding of cellular proteins to guarantee their function or by advancing the degradation of terminally misfolded proteins to prevent damage.^234^ With advancing age, the capability of this proteostasis supporting network tends to weaken, which facilitates the progression of neurodegenerative diseases. In general, two key hypotheses exist explaining for polyQ expansions may trigger cellular dysfunction: (i) neurotoxicity stems from the ability of polyQ-expanded proteins to recruit other vital cellular proteins into the aggregates; (ii) aggregating polyQ proteins partly inhibit the ubiquitin–proteasome system for protein degradation.^235^ These two models are not exclusive but may act in concert. Overall, protein misfolding and aggregation are prevented by the machinery of molecular chaperones. Some chaperones such as the Hsp70 family members also modify polyQ aggregation and inhibit its toxicity. These results point out the functional relationship between molecular chaperones, the ubiquitin–proteasome system, and polyQ aggregation.^235^ Chaperone therapy is a recently developed therapeutic strategy against protein misfolding diseases. Molecular chaperones utilizing the heat shock protein and other chaperone proteins have been reported able to handle abnormally accumulated proteins as a new approach to neurodegenerative diseases.^236^ Understanding how chaperones interrelate to disease progression is essential for the advancement of therapeutic strategies to combat these debilitating diseases.^234^

**Disease models** constitute another widely explored concept in the polyQ disease-related documents in the CAS Content Collection (Figure 10). PolyQ diseases affect neural tissue, which is particularly hard to obtain from patients. Therefore, cellular and organism models are essential for successful research in this area. Cellular models are indispensable element in research and have significant contributions to the discovery and validation of multiple pathological changes related to polyQ diseases. A variety of cellular models include (i) fibroblasts extracted from patients by a skin biopsy;^237,238^ (ii) embryonic stem cells, which contain disease-associated genetic patterns and can be further differentiated into any cell in the human body;^239^ (iii) induced pluripotent stem cells comprising patient-specific genetic information, dividing unlimitedly and able to be differentiated into any disease- related cell populations, including neurons;^240,241^ (iv) human embryonic kidney 293 (HEK 293T) cells having the advantage of simple transfection and high-level transgene expression;^242^ and (v) yeast cell models, inexpensive and appropriate for large-scale genetic and pharmacological screening.^45^ Further, animal models exhibiting more advanced phenotypes and typical behaviors are an essential requisite for polyQ disease modeling. Simple model organisms include the nematode *Caenorhabditis elegans*, the fruit fly *Drosophila melanogaster*, and the zebrafish *Danio rerio*. They helped verify pathogenic features of polyQ diseases such as the aggregate formation, the mutant proteins toxicity, the neurotransmission deficiencies and the progressive neuronal degeneration.^45^ Rodent models have been successfully used in studying behavioral phenotypes of Huntington’s disease.^243^ Recently, large mammalian models of Huntington’s disease and SCA3 in monkeys, marmosets, minipigs, pigs, and sheep have been generated by using genome-editing technology. The advancement of modern gene-editing technologies, such as meganucleases, zinc finger nucleases (ZFNs), transcription activator-like effector nucleases (TALENs) and especially the CRISPR-Cas9 technique, has a particular value for the generation of relevant polyQ models, which have substantially advanced the research process.^45^

**Stem cells** are also a commonly explored concept in the polyQ disease-associated documents in the CAS Content Collection (Figure 10). One of the novel therapeutic approaches for treating polyQ diseases focuses on the development of cell replacement therapies.^244^ Such therapies are anticipated in either replacing damaged neurons or stimulating the endogenous neurogenesis pathways of the brain. Stem cells represent a favorable tool for regenerative medicine in human disorders cure. Successful stem-cell transplantation attempts in models of polyQ diseases have been made and believed to hold great promise for the advancement of new cell-based therapies for polyQ diseases.^244^

**Histone deacetylase (HADC) enzymes** are known to remove acetyl group from lysine residue of histones and other proteins. In neurodegenerative diseases, histone acetylation homeostasis is significantly compromised, giving rise to hypoacetylation. In particular, it has been evidenced that the chromatin acetylation status is critically impaired in polyQ disorders. Therefore, histone hyperacetylation triggered by inhibition of HDACs has neuroprotective effect and HDAC inhibitors have been suggested as a relevant treatment approach.^245^ Small molecule inhibitors of HDACs notably affect neuronal differentiation and neurite outgrowth, and thus exhibit a potential as therapeutic agents for treatment of neurodegenerative diseases including polyQ diseases.^246^ These inhibitors of the zinc-dependent classes of HDACs belong to 4 classes regarding their chemical structure: hydroxamates, cyclic peptides, short chain fatty acids and benzamides.^247^ The clinical application of these broadly acting compounds for neurodegenerative disorders is limited however by their toxicity. Moreover, additional studies are needed to fully understand the mechanisms associated with the beneficial effects of selective HDAC inhibitors, to identify specific substrates and to further define the pathways in which specific HDAC enzymes are involved.

A rational approach to treating polyQ diseases is to suppress production of the mutant protein ahead of its deleterious effects. Therefore, silencing the mutant gene would be of therapeutic benefit. **Gene silencing** therapies have been developed, using either RNA interference (RNAi) or antisense oligonucleotide (ASO) strategies.^248^ Both have been proven promising in studies in animal models of polyQ diseases, including SBMA, SCA1, and HD.^249^ A number of challenges must be dealt with, however, before these results can be translated to the clinic.

**Figure 11:**
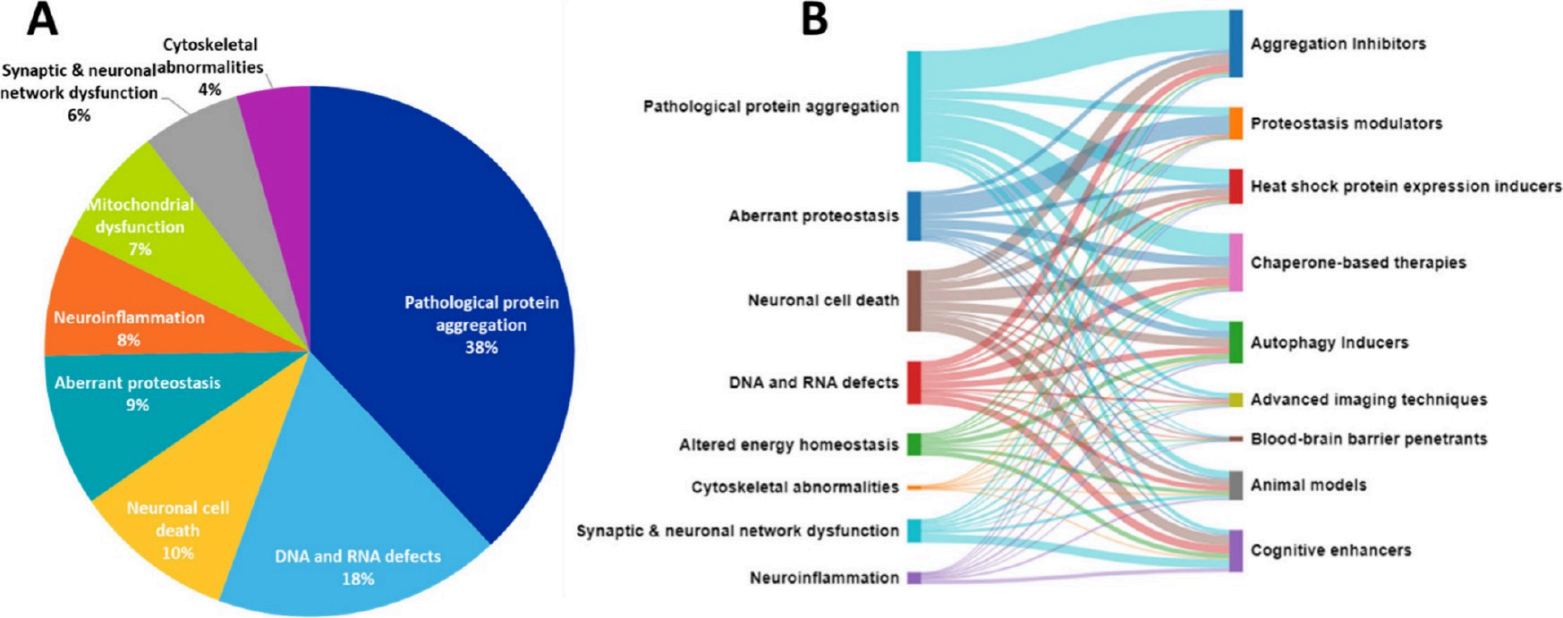
Distribution of documents related to polyQ disease hallmarks in CAS Content Collection (A) and their co-occurrence with various disease remedies (B).

The distribution of documents in the CAS Content Collection related to the polyQ disease hallmarks concepts are presented in Figure 11A, while a Sankey graph of the number of documents in which these concepts co-occur with certain disease remedies are shown in Figure 11B. Pathological protein aggregation appears as the most widely discussed attribute of the polyQ diseases, it also co-occurs in documents related to the majority of therapeutic approaches. This is an anticipated observation since protein aggregation is a key pathological hallmark of the majority of neurodegenerative diseases and is in fact related to virtually all other neurodegeneration attributes. Understandably, it most often co-occurs with the aggregation inhibitors as a therapeutic strategy. Chaperone-based therapies also appear as closely targeted to pathological protein aggregation. Neuronal cell death and the aberrant proteostasis are another polyQ disorder attributes discussed along with multiple disease remedies (Figure 11B). Proteostasis modulators along with the chaperone-based therapies are most frequently targeted to aberrant proteostasis. Neuronal cell death is about equally targeted by several therapeutic strategies: aggregation inhibitors, chaperone-based therapies, and cognitive enhancers. Cognitive enhancers seem like the major strategy toward synaptic and neuronal network dysfunction.

**Figure 12:**
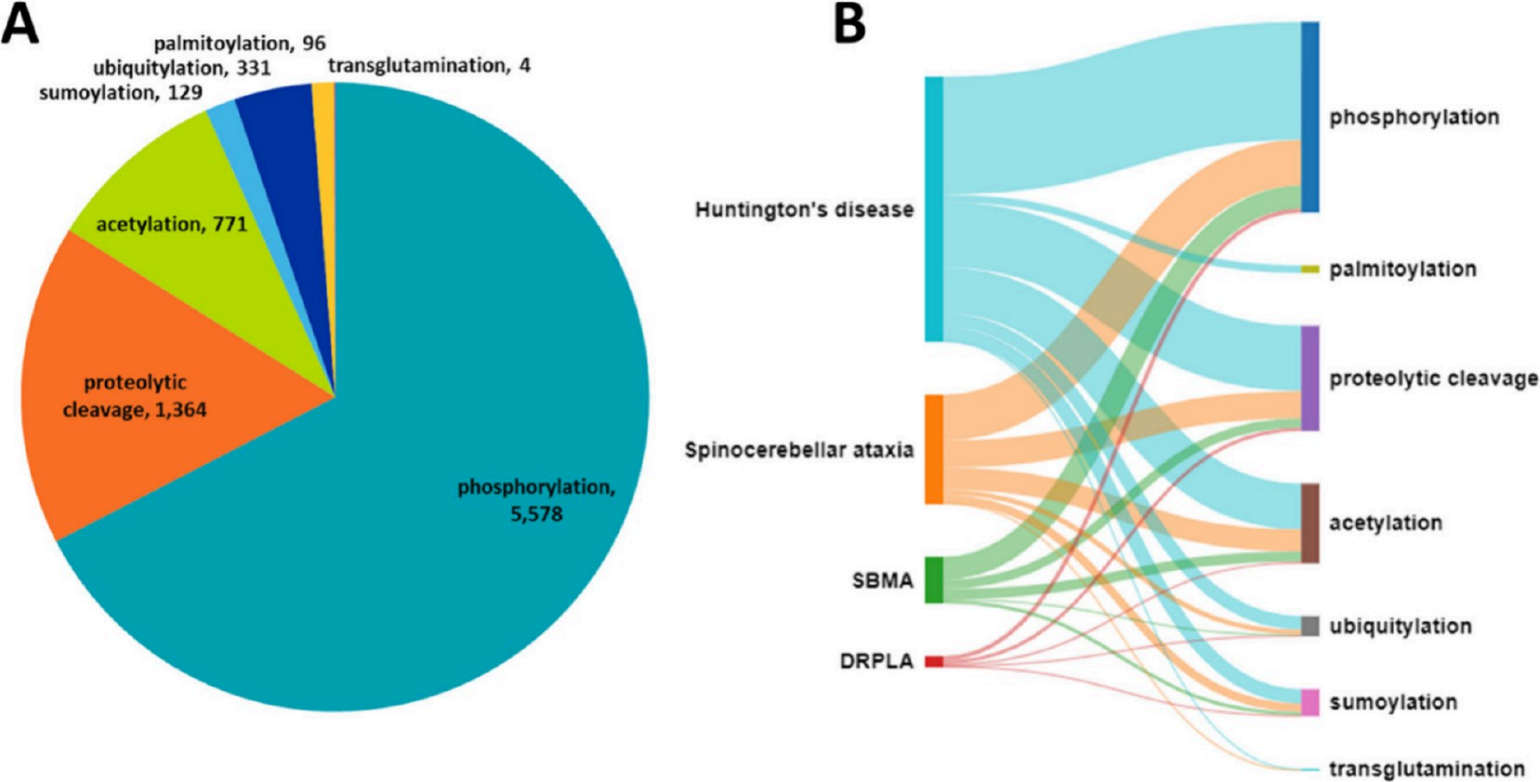
Distribution of documents related to polyQ disease-associated post-translational modifications related to CAS Content Collection (A) and their co-occurrence with the various polyQ diseases (B).

Research has revealed that post-translational modifications of the polyglutamine proteins are involved in their neurotoxicity and can significantly modulate it. The distribution of documents in the CAS Content Collection discussing various types of post-translational protein modifications is presented in Figure 12A, and the co-occurrence of these terms with the various members of the polyQ diseases are shown in Figure 12B. Generally, Huntington’s disease, which is the subject of the largest number of documents in this subset, co-occurs also with the largest number of documents discussing post-translational modifications. On the other hand, phosphorylation, as the major type of post-translational modification, is related to all kinds of polyQ diseases. The largest part of documents discuss phosphorylation, in association with Huntington’s disease. Palmitoylation only co-occurs with Huntington’s disease-related documents, while transglutamination–with Huntington’s disease and spinocerebellar ataxias.

**Figure 13:**
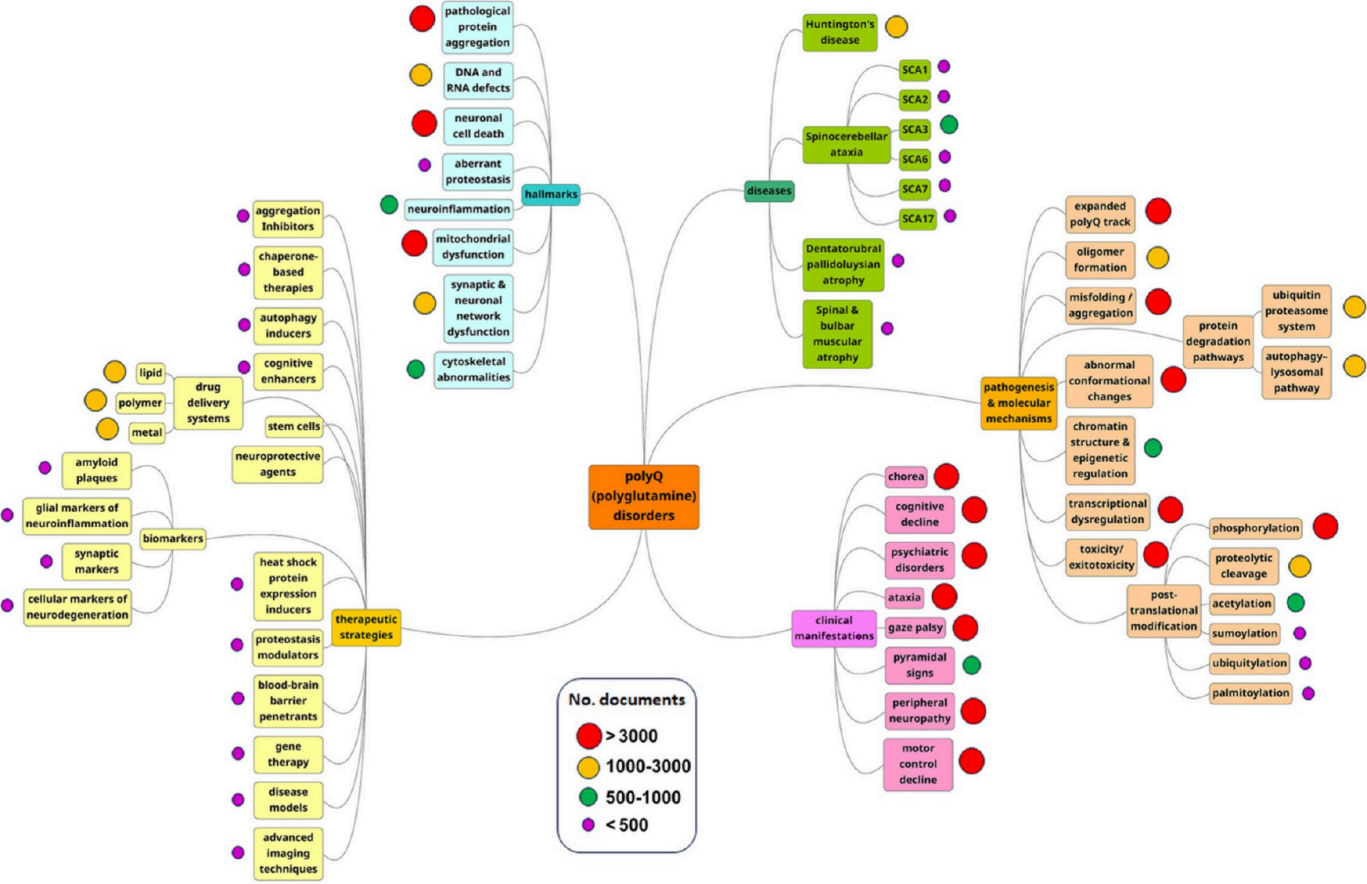
Mind map of the polyQ disease research area with indication of the number of documents in each subcategory.

In Figure 13 we present a mind map of the polyQ disease research area, with indication of the number of documents related to each subcategory. The pathogenesis and molecular mechanisms, and the clinical manifestations of diseases are the areas attracting most attention.

## Notable Recent Patents

Table 3 below summarizes exemplary notable patents related to polyQ disease. These examples were selected by CAS subject matter experts to highlight the range of discussed materials and applications in treatment of the polyQ diseases.

**Table 3:** Notable patent application publications in the field of polyQ disease in recent years

**Patent Number**	**Publication Year**	**Patent Assignee**	**Title**	**Details**
WO2022221276	2022	University of Pennsylvania	Compositions useful for treatment of Spinal and Bulbar Muscular Atrophy (SBMA)	Recombinant adeno-associated virus (rAAV) vector having an AAV capsid and a vector genome comprising a sequence encoding at least one hairpin forming miRNA that comprises a targeting sequence which binds a target site on the mRNA of human androgen receptor, wherein the miRNA inhibits expression of human androgen receptor. Compositions containing a rAAV vector and methods of treating SBMA in patients comprising administration of a rAAV vector.
WO2023250316	2023	PTC Therapeutics	Synthesis of thienopyridines for treating spinocerebellar ataxia type 3 (Machado-Joseph disease)	Synthesis of thienopyridines for improving pre-mRNA splicing in a cell; 2-((1*S*,2*S*)-2-Aminocyclopentyl)-3,5-dichloro-N-(thien-2-ylmethyl)thieno[3,2-b]pyridin-7-amine, and related compounds and pharmaceutical compositions can treat or ameliorating spinocerebellar ataxia type 3, also known as Machado-Joseph disease.
WO2023250325	2023	University of California	Preparation method of compositions for treating Huntington’s disease	Provided are methods for reducing the level of an RNA transcript produced from an mHTT allele in an allele-specific manner, as well as systems and compositions for carrying out the methods.
WO2023244682	2023	Design Therapeutics	Methods and compounds for modulating inherited genetic diseases	Provided are transcription modulator mol. compounds, compositions, and methods of treating various genetic diseases including spinocerebellar ataxias and Huntington’s disease.
WO2023230282	2023	The General Hospital Corporation	Modulation of BACE1 as a therapy for spinocerebellar ataxia	Provided are methods and compositions for treating neurodegenerative diseases including Spinocerebellar Ataxia comprising administering a BACE1 inhibitor.
WO2023239747	2023	University of Utah	Method containing cardiac glycoside and topoisomerase inhibitor for modulating ATXN2 expression in cell	A method of modulating ATXN2 expression in a cell comprising administering to the cell an effective amount of an ATXN2 modulating agent selected from the group consisting of a cardiac glycoside, an HSP90 inhibitor, an NaK-ATPase inhibitor, a topoisomerase inhibitor, or a combination thereof.
WO2023225506	2023	University of North Carolina at Chapel Hill	Compositions and methods comprising synthetic RNA molecules for treatment of intragenic nucleotide repeat disorders	A synthetic RNA molecule comprising an antisense strand, complementary to a portion of the nucleotide sequence of a mammalian gene comprising an intragenic nucleotide repeat region wherein the nucleic acid molecule degrades and/or inhibits the expression of the mammalian gene mRNA. A method for treating Huntington’s disease or myotonic dystrophy.
WO2023170115	2023	F. Hoffmann-La Roche	Pyrido[1,2-a]pyrimidin-4-one derivatives	Compounds that reduce the protein level of huntingtin and which are useful in the treatment of Huntington’s disease.
WO2024026061	2024	Biogen	Compounds for treating Huntington’s disease	A compound for treating a disorder in which lowering mutant huntingtin protein in a subject is of therapeutic benefit, specifically in treating Huntington disease. This disclosure also features a composition containing the same as well as methods of using and making the same.
WO2024010818	2024	University of Tennessee	Proteolysis targeting chimera (PROTAC) of selective androgen receptor degrader (SARD) compounds and methods of use thereof	PROTAC of SARD compounds which are useful in treating among others, spinal and bulbar muscular atrophy, and pathogenic polyglutamine polymorphisms of androgen receptor in a subject.

## Commercial Preclinical Development

Almost 45 substances are being researched and developed preclinically for the treatment of polyglutamine diseases. The vast majority of these compounds are for the treatment of Huntington’s disease (HD) but substances for the treatment of spinocerebellar ataxias (SCA) are also in the development pipeline (Table 4). Table 4 highlights drugs in preclinical development for the treatment of polyQ diseases, their class, mechanism of action, and the companies developing them. A wide range of therapies are being investigated including small molecules, protein degraders, RNA therapeutics, gene therapy, cell therapies, among others. The top five preclinical researched drug classes for the treatment of polyQ diseases include small molecule drugs followed by gene therapy, RNA interference agents, protein degraders, and stem cell therapy.

**Table 4:** Drugs for PolyQ diseases in commercial preclinical development

**Drug**	**Drug class**	**Mechanism**	**PolyQ disease indication**	**Company, location**
AJ-201^250^	Neuroprotectant	Transcription factor Nrf2 stimulant	HD, SCA, SBMA (Phase 1/2)	Avenue Therapeutics, USA
ALN-HTT^251^	RNA interference	Gene expression inhibitor	HD	Alnylam, USA
ALN-HTT02^252^	RNA interference	Gene expression inhibitor	HD	Alnylam, USA
Anima Huntingtin translation inhibitor^253^	mRNA therapy	Gene expression inhibitor	HD	Anima Biotech, USA; Takeda, Japan
ASK-005^254^	Cognition enhancer	Arachidonic acid inhibitor	HD	ASDERA, USA
ATLX-1095^255^	Monoclonal antibody, human	HTT inhibitor	HD	Alchemab Therapeutics, UK
Debamestrocel^256^	Stem cell therapy	Glial cell derived neurotrophic growth factor agonist	HD	BrainStorm Cell Therapeutics, USA
ET-101^257^	Gene therapy	Caveolin stimulant	HD	Eikonoklastes Therapeutics, USA
HB AdMSC^258^	Stem cell therapy, cognition enhancer	Undisclosed	HD	Hope Biosciences, USA
Huntington’s disease therapy^259^	Undisclosed	Undisclosed	HD	Aitia, USA; UCB, Belgium
Huntington’s disease therapy^260^	PROTAC	HTT inhibitor, E3 ubiquitin ligase stimulant, protein degrader	HD	Arvinas, USA
Huntington’s disease therapy^261^	RNA interference	Gene expression inhibitor	HD	Atalanta Therapeutics, USA; Biogen, USA
Huntington’s disease therapy^262^	Neuroprotectant	Undisclosed	HD	BPGbio, USA
Huntington’s disease therapy^263^	Gene therapy	DNA editing, CRISPR	HD	HuidaGene Therapeutics, China
Huntington’s disease therapy^264^	Antisense oligonucleotide therapy	Undisclosed	HD	Ionis, USA; Roche, Switzerland
Huntington’s disease therapy^265^	Neuroprotectant	DNA damage repair	HD	LoQus23 Therapeutics, USA
Huntington’s disease therapy^266^	Neuroprotectant, cognition enhancer	Reverses mitochondrial dysfunction	HD	MitoRx Therapeutics, UK
Huntington’s disease therapy^267^	RNA interference	Utilized a small hairpin RNA or short hairpin RNA for gene expression inhibition	HD	Novartis, Switzerland; Voyager Therapeutics, USA
Huntington’s disease therapy^268^	Gene therapy	Deliver functional gene utilizing AAV vector	HD	Passage Bio, USA
Huntington’s disease therapy^269^	Gene therapy	Genome editing	HD	Prime Medicine, USA
Huntington’s disease therapy^270^	Monoclonal antibody	Targeting protein RACK-1, Protein kinase C inhibitor	HD	ProMIS Neurosciences, USA
Huntington’s disease therapy^271^	Stem cell therapy	Mesenchymal Stem Cells	HD	Trailhead Biosystems, USA
IC 100–05^272^	Anti inflammatory	Inflammasome ASC Inhibitor	HD	ZyVersa Therapeutics, USA
INT41^273^	Gene therapy	mHTT protein inhibitor, selectively binds to mHTT protein	HD	Vybion, USA
M102^274^	Neuroprotectant	Activates Nrf2 and HSF1	HD	Aclipse Therapeutics, USA
NP-001^275^	Neuroprotectant, Cognition enhancer	Oxidizing agent	HD	Neuvivo, USA; Neuraltus, USA
NT-0100^276^	Antisense oligonucleotide therapy	Gene expression inhibitor	HD	NeuBase Therapeutics, USA
NXL-002^277^	Gene therapy	Regenerates neurons	HD	NeuExcell Therapeutics, USA; Spark Therapeutics, USA
OCCT-HTT siRNA^278^	RNA interference	Gene expression inhibitor; HTT inhibitor	HD	Ophidion, USA
ORI-113^279^	Protein degrader	HTT protein degrader	HD	Origami Therapeutics, USA
ORI-503^279^	Protein conformation correctors	HTT proteins conformation corrector	HD	Origami Therapeutics, USA
ReS18-H^280^	Cognition enhancer	Restores function and improve survival of medium spiny neurons leading to reactivation of corticostriatal transmission	HD	reMYND, Belgium
SC-379^281^	Stem cell therapy	Glial progenitor cells	HD	Sana Biotechnology, USA
SCA3 therapy^282^	Undisclosed	Undisclosed	SCA3	PTC Therapeutics, USA
SLS009^283^	Protein degrader	Protein targeted autophagy	HD	Seelos Therapeutics, USA
SMDG-HD11^284^	RNA therapy	Undisclosed	HD	S. M. Discovery Group, UK
SOL175^285^	Gene therapy	Reduces abnormally folded protein	HD	SOLA Biosciences, USA
SOL176^285^	Gene therapy	Reduces abnormally folded protein	HD	SOLA Biosciences, USA
TAK-686^286^	Gene therapy	Zinc finger nucleases can target regions of DNA to modify them or stop RNA from being made.	HD	Sangamo Therapeutics, USA; Takeda, Japan
TQS-168^287^	Neuroprotectant	PPARG coactivator 1 alpha agonist	HD	Tranquis Therapeutics, USA
TT-P34^288^	Peptide therapy	Activates pathways that can bypass mHTT to reactivate CREB	HD	Teitur Trophics, Denmark
VTX-003^289^	Antibody therapy	mHTT protein inhibitor, selectively binds mutant HTT (and not normal HTT) to clear mutant HTT	HD	VectorY Therapeutics, Netherlands

## Clinical Trials

**Figure 14:**
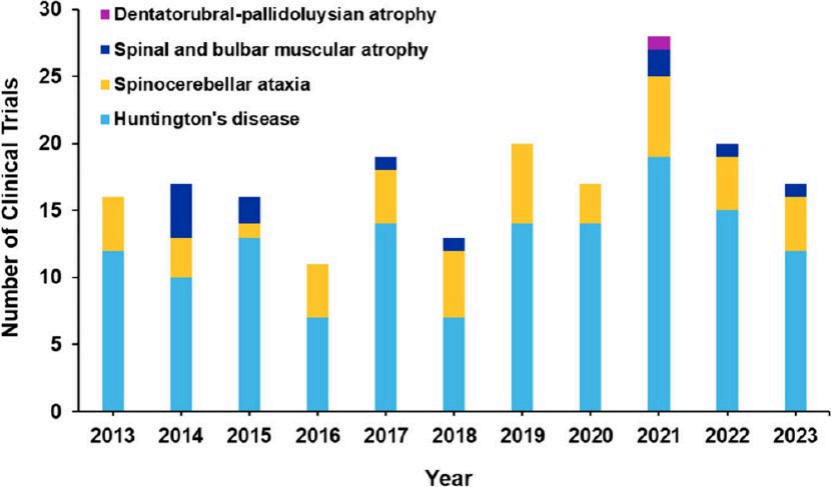
Number of polyglutamine disease clinical trials by year.

Clinical Trials researching the treatment of polyglutamine diseases are explored in this section to gain an overall view of the past and current state of clinical development. Around 200 clinical trials have been registered on clinicaltrials.gov over the last 10 years for polyQ diseases, reinforcing not only their low numbers in clinical development but also the rarity of these conditions. Figure 14 shows an oscillating curve equaling 17 to 30 clinical trials per year for polyQ diseases combined, between the years 2013 to 2023. Huntington’s disease dominates in numbers for yearly clinical trials followed by Spinocerebellar ataxia (SCA), Spinal and bulbar muscular atrophy (SBMA), and Dentatorubral-pallidoluysian atrophy (DRPLA).

**Figure 15:**
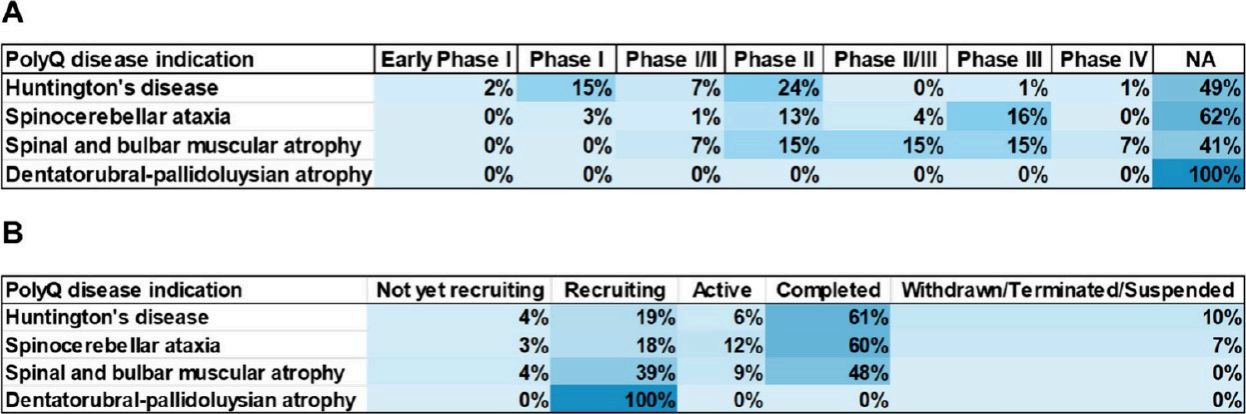
Percentage of polyglutamine disease clinical trials in various: (A) phases; (B) statuses.

Analysis of PolyQ disease clinical trials reveals that nearly half of all trials for the different indications are not phased (Figure 15A). The phase that contains the next largest group of trials is Phase II studies for Huntington’s disease (HD), Phase III studies for spinocerebellar ataxia (SCA), and both Phase II and Phase III studies for spinal and bulbar muscular atrophy (SBMA). The Dentatorubral-pallidoluysian atrophy (DRPLA) indication contains one clinical trial (NCT05489393) on clinicaltrials.gov which is a global patient registry to establish a database of patient-reported data on individuals affected with DRPLA from around the world. Nearly half of all clinical trials for HD, SCA, and SBMA have been completed (Figure 15B). The status with the next largest group of trials is the recruiting status with is encouraging as new clinical trials are created and carried out to research the treatment of these rare polyglutamine diseases, offering hope to patients worldwide.

Finally, representative clinical trials examining polyQ disease therapeutics are highlighted in Table 5 categorized by therapy type and disease indication. These are examined in further detail below to showcase a variety of therapeutic strategies, interventions, and targeted conditions in clinical development along with their status, phase, and any published results.

**Table 5:** Highlighted PolyQ disease clinical trials

**Therapy type**	**PolyQ disease indication**	**Intervention**	**Sponsor, location**	**Status**	**Phase**	**NCT Number**
Antisense oligo-nucleotide	Huntington’s disease	Tominersen	Hoffmann-La Roche, Switzerland	Recruiting	Phase II	NCT05686551
				Completed	Phase III	NCT03761849
Antisense oligo-nucleotide	Huntington’s disease	WVE-003	Wave Life Sciences, USA	Recruiting	Phase I/Phase II	NCT05032196
				Active, not recruiting	Phase II/Phase III	NCT04219241
				Not yet recruiting	Phase III	NCT06097780
Computer based cognitive stimulation	Huntington’s disease	Virtual reality computer simulation	Santa Creu Hospital, Spain	Active, not recruiting	NA	NCT05769972
Small molecule	Huntington’s disease	PTC518	PTC Therapeutics, USA	Recruiting	Phase II	NCT05358717
Small molecule	Huntington’s disease	SAGE-718	Sage Therapeutics, USA	Recruiting	Phase II	NCT05107128
					Phase II	NCT05358821
					Phase III	NCT05655520
Stem cell therapy	Huntington’s disease	NestaCell	Azidus, Brazil	Active, not recruiting	Phase I	NCT02728115
				Active, not recruiting	Phase II/Phase III	NCT04219241
				Not yet recruiting	Phase III	NCT06097780
Antisense oligonucleotide	SCA1, SCA3, and Huntington’s disease	VO659	Vico Therapeutics, Netherlands	Recruiting	Phase I/Phase II	NCT05822908
Small molecule	SCA3	Trehalose	National University of Malaysia	Recruiting	NA	NCT04399265
			National University of Malaysia	Completed	NA	NCT04426149
			Seelos Therapeutics, USA	Active, not recruiting	Phase II/Phase III	NCT05490563
Small molecule	SCA1, SCA2, SCA3, SCA6, and SCA7	Troriluzole	Biohaven Pharmaceuticals, USA	Active, not recruiting	Phase II/Phase III	NCT02960893
					Phase III	NCT03701399
Stem cell therapy	SCA1, SCA2, SCA3, and SCA6	Umbilical cord mesenchymal stem cell	Sclnow Biotechnology, China	Not yet recruiting	Phase II	NCT03378414
Small molecule	Spinal and bulbar muscular atrophy	AJ201	AnnJi Pharmaceutical, Taiwan	Completed	Phase I	NCT04392830
				Active, not recruiting	Phase I/Phase II	NCT05517603
Small molecule	Spinal and bulbar muscular atrophy	Clenbuterol	Padova University Hospital, Italy	Not yet recruiting	Phase II	NCT06169046
Patient registry	Dentatorubral-pallidoluysian atrophy	Global patient registry	CureDRPLA, USA	Recruiting	NA	NCT05489393

Antisense oligonucleotide (ASO) therapeutics in clinical trials for the treatment of HD include tominersen (NCT05686551) and WVE-003 (NCT05032196). After halting a Phase III clinical trial (NCT03761849), post hoc analysis revealed tominersen may benefit young adult patients with lesser disease burden.^290^ A new Phase II clinical trial (NCT05686551) was created to research this finding and is currently recruiting participants. Interim results for Phase I/Phase II clinical trial NCT05686551, reveals that a single dose of another ASO, WVE-003 (30 or 60 mg), led to a mean 35% reduction in mHTT in the cerebrospinal fluid compared to a placebo. More upcoming trial findings are expected by June 2024.^291^ Another treatment of Huntington’s disease is examined with clinical trial NCT05769972. Researchers at Santa Cre Hospital in Spain are recruiting for their study (NCT05769972) to research the use of a computer based cognitive rehabilitation program in patients with HD with expectations that the program will have a greater beneficial effect on the cognitive status of patients compared to control modalities such as music therapy.^292^

Sage Therapeutics’ and PTC Therapeutics’ small molecule drugs PTC518 and SAGE-718 are currently recruiting for their Phase II and Phase II/Phase III clinical trials, respectively. PTC518 modifies RNA splicing, disrupting the production of all HTT protein forms, while SAGE-718 is a NMDA receptor positive allosteric modulator. Clinical trials (NCT05358717, NCT05107128, NCT05358821, and NCT05655520) will research the safety, tolerability, and efficacy of these drugs for the treatment of HD. A previous Phase I clinical trial for PTC518 revealed the drug reduced HTT mRNA in a dose-dependent manner.^293^ SAGE-718 has completed initial single and multiple ascending dose clinical studies, where it demonstrated efficacy in disease-relevant populations.^294^ In addition, SAGE-718 was granted both FDA Fast track designation^295^ in 2022 and FDA Orphan Drug Designation in 2023.^296^

Phase I and Phase II/Phase III clinical trials (NCT02728115 and NCT04219241) assessing the safety and efficacy of Cellavita’s NestaCell, a stem cell therapy derived from immature human dental pulp, are currently active for the treatment of Huntington’s disease. Another Phase III clinical trial (NCT06097780) researching NestaCell is not yet recruiting but has an estimated start date of June 2024. A previous Phase I clinical trial revealed no serious adverse events and improved HD motor manifestations for the treatment of HD with NestaCell.^297^

Clinical trials researching the treatment of Spinocerebellar ataxia (SCA) are also examined. Currently recruiting participants, Phase I/Phase II clinical trial NCT05822908 will evaluate the safety and tolerance of four different doses of ASO VO659 in people with SCA1, SCA3, and Huntington’s disease. The study will measure concentrations of VO659 in cerebral spinal fluid and blood after single and multiple doses.^298^ Small molecule, trehalose, is active with a Phase II/Phase III clinical trial (NCT05490563) to measure its safety and efficacy for the treatment of SCA3. Trehalose has also received FDA orphan drug designation.^299^ Another, small molecule drug troriluzole is currently researched in Phase II and Phase III trials (NCT02960893 and NCT03701399) examining its efficacy at 140 mg once daily in subjects with SCA. Troriluzole inhibits voltage gated sodium channels and reduces synaptic glutamate.^300^ Unfortunately, results showed no improvement in patients on troriluzole except for a subset with SCA3, which prompted a new drug application to the US FDA.^301^ Troriluzole has previously received Fast-Track and Orphan drug designation from the FDA for the treatment of SCA.^301^ Lastly, Sclnow biotechnology is not yet recruiting but will be researching their Umbilical Cord Mesenchymal Stem Cell therapy (UC-MSC) for patients with spinocerebellar ataxia.^302^ The Phase II clinical trial (NCT03378414) will not only verify safety and efficacy but will also research the possible mechanisms of UC-MSC therapy in SCA.

For the treatment of Spinal and Bulbar Muscular Atrophy (SBMA), preliminary pilot studies reveal small molecule clenbuterol is effective at improving motor function in SBMA.^303^ Research will continue with Phase II clinical trial NCT06169046 soon, as it is not yet recruiting, to research the safety and efficacy of clenbuterol as a treatment for SBMA. Another small molecule, AJ201 has been granted FDA orphan drug designation for the treatment of SBMA, HD, and SCA. A Phase I safety study in healthy volunteers was successfully completed in 2021.^250^ A Phase I/Phase II study (NCT05517603) is currently active evaluating safety, tolerability, pharmacokinetics, and pharmacodynamics of AJ201 for the treatment of SBMA. Lastly Dentatorubral-pallidoluysian atrophy (DRPLA) only has one registered clinical trial.^304^ This trial (NCT05489393) will establish a global patient registry consisting of a database with patient reported data on individuals affected with DRPLA from around the world.

## Outlook and Perspectives

There is ongoing research aimed at developing disease-modifying therapies for polyQ diseases. Approaches include gene silencing techniques like RNA interference (RNAi) or antisense oligonucleotides (ASOs), small molecule inhibitors targeting toxic protein aggregation, and strategies to enhance protein degradation pathways. Advancements in genetic testing and understanding of disease mechanisms have paved the way for personalized treatment approaches tailored to individual patients’ genetic profiles and disease progression. Research is focusing on identifying compounds and interventions that can protect neurons from the toxic effects of mutant polyQ proteins, potentially slowing disease progression.

Stem cell-based approaches hold promise for replacing damaged neurons or providing trophic support to degenerating neurons in polyQ diseases. Researchers are investigating the potential of stem cell transplantation as a therapeutic avenue.

Identifying reliable biomarkers for disease onset, progression, and response to treatment is crucial for developing clinical trials and monitoring therapeutic efficacy. Advances in imaging techniques, biofluid analyses, and molecular biomarkers are ongoing areas of investigation.

Further elucidating the molecular mechanisms underlying polyQ diseases, including protein aggregation, cellular toxicity, and dysfunction of intracellular pathways, will provide valuable insights for therapeutic development. Roadblocks complicating the polyQ disease research advances are listed in Table 6.

**Table 6:** Roadblocks in the polyQ diseases research:

**Roadblock**	**Details**
Complex pathophysiology	The pathophysiology of polyQ diseases involves intricate molecular mechanisms, including protein misfolding, aggregation, and toxicity, as well as dysregulation of cellular processes. Understanding these complexities presents a significant challenge.
Variable clinical presentation	PolyQ diseases exhibit significant heterogeneity in clinical presentation, age of onset, and disease progression, even among individuals with the same genetic mutation. This variability complicates diagnosis, prognosis, and the development of targeted therapies.
Limited disease models	Animal models, such as transgenic mice expressing mutant polyQ proteins, have provided valuable insights into disease mechanisms. However, these models do not fully recapitulate the human disease phenotype, limiting their utility for drug discovery and translational research.
Blood-brain barrier penetration	Many potential therapeutics for polyQ diseases may face challenges in crossing the BBB to reach target neurons in the central nervous system (CNS). Strategies to enhance BBB penetration while maintaining therapeutic efficacy and minimizing off-target effects are needed.
Lack of biomarkers	The identification of reliable biomarkers for disease diagnosis, prognosis, and monitoring treatment response is essential for clinical trials and personalized medicine approaches. However, validated biomarkers for polyQ diseases remain limited, hampering disease management and therapeutic development.
Limited treatment options	Currently, there are no disease-modifying treatments for polyQ diseases, and symptomatic therapies offer only partial relief of symptoms. Developing effective therapies that can slow or halt disease progression remains a significant challenge.
Ethical considerations	Emerging gene-editing technologies, such as CRISPR-Cas9, hold promise for correcting disease-causing mutations in polyQ diseases. However, ethical considerations surrounding the use of these technologies, including off-target effects and germline editing, must be carefully addressed.

Future research directions in the field of the polyQ disease research need to be concentrated on:

- Developing novel strategies to prevent or disaggregate toxic protein aggregates, including targeting specific steps in the aggregation process or enhancing cellular clearance mechanisms.
- Investigating the factors contributing to the variable clinical presentation and progression observed in polyQ diseases, including genetic modifiers, environmental influences, and cellular context.
- Investigating the role of non-neuronal cells, such as glia and immune cells, in disease pathogenesis and progression, and exploring potential therapeutic targets in these cell types.
- Exploring the potential synergistic effects of combining different therapeutic approaches, such as targeting protein aggregation alongside neuroprotective or anti-inflammatory strategies.
- Designing clinical trials with robust end points, appropriate patient stratification, and innovative trial designs, such as adaptive or platform trials, to accelerate the translation of promising therapies to patients.

While significant challenges remain, continued research efforts hold promise for advancing our understanding of polyQ diseases and developing effective treatments to improve patients’ quality of life and prognosis.
